# Novel naphthylpyridines from cobalt-catalyzed cyclotrimerization of a chiral diyne

**DOI:** 10.1007/s00706-017-2083-9

**Published:** 2017-11-28

**Authors:** Volkmar Trommer, Fabian Fischer, Marko Hapke

**Affiliations:** 1 Leibniz-Institut für Katalyse an der Universität Rostock (LIKAT), Albert-Einstein-Strasse 29a, 18059 Rostock, Germany; 2 Institut für Katalyse (INCA), Johannes Kepler Universität Linz, Altenberger Strasse 69, 4040 Linz, Austria

**Keywords:** Biaryls, Cycloadditions, Catalysis, Heterocycles, Alkynes

## Abstract

**Abstract:**

The concise synthesis of a novel chiral diyne substrate for the assembly of chiral naphthylpyridines was described and different conditions for the cobalt-catalyzed co-cyclotrimerization with nitriles investigated. The products are novel naphthylpyridines possessing configurationally stable biaryl axes.

**Graphical abstract:**

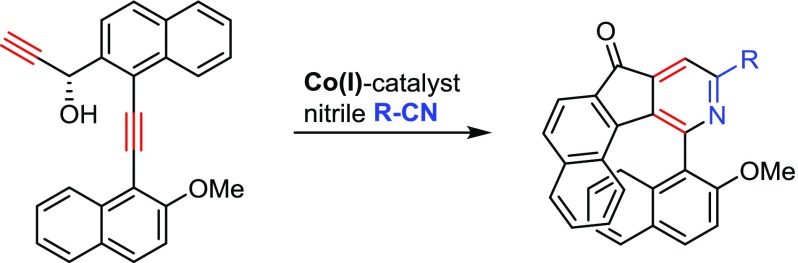

**Electronic supplementary material:**

The online version of this article (doi:10.1007/s00706-017-2083-9) contains supplementary material, which is available to authorized users.

## Introduction

Helicenes and axially chiral biaryls constitute two kinds of chirality which are manifested in many structurally fascinating examples. Chiral and achiral biaryl compounds were found or play a role in many natural products [1–3], in drug discovery [4, 5], or as a key building block of many chiral ligands for stereoselective catalysis [6–8], to name a few examples. The arsenal of synthetic methods for the atroposelective synthesis of biaryls has become extremely broad especially during the recent two decades [9]. Helicenes have found application in many similar areas of application due to their unique helical chirality as well as rigid conjugated structures and different approaches for their synthesis have been explored [10–13]. Combining the ease of functional group introduction and modification in the biaryl backbone with elements contributing to the large configurational stability of many higher helicenes can be beneficial to the construction of molecular ligand platforms. An attractive and in recent years significantly developed access to helicenes and biaryls was found in the application of the [2 + 2 + 2] cycloaddition reaction [14]. Especially intramolecular transformations of di-, tri-, and oligoalkynes were developed as a systematic access, employing a wide range of transition metal catalysts [15]. The assembly of chiral helicenes by cyclotrimerization reactions was realized with different metal catalysts [16–18].

We have investigated the asymmetric synthesis of naphthyl isoquinolines by [2 + 2 + 2] cycloaddition of diynes and nitriles applying chiral indenyl Co(I)-catalysts, especially also under photochemical conditions [19, 20]. The construction of naphthyl pyridines with annellated, unsymmetrical five-membered rings by the Co(I)-mediated cyclotrimerization of the corresponding diynes and nitriles was essential during an investigation on epimerization barriers of this class of compounds [21]. Application of the chiral indenyl Co(I)-complex mentioned above in the synthesis of tetrahydro [6] helicene from triynes was investigated by us and the group of Stará as well [22].

In the present work, we planned to exploit our synthetic experience in the synthesis of alkyne derivatives and aimed at synthesizing chiral diynes as precursor molecules for highly sterically hindered diastereomeric naphthyl pyridine atropisomers, which should be separable by e.g. chromatography on silica gel. The following section describes the synthesis of the substrates and surprising results for the cyclotrimerization step.

## Results and discussion

The work was initiated with the planning of an efficient synthetic access for the diyne substrate leading to the envisioned product **I**. In Scheme 1 the retrosynthetic analysis is shown. The synthesis would start from the two halogenated naphthalene building blocks **1** and **2** from where subsequent introduction of the alkyne moieties will lead to substituted alkyne **4** and subsequently diynes **8** and **9**. The final step will be a transition metal-catalyzed [2 + 2 + 2] cycloaddition reaction with a nitrile, leading to the finally assembled product **I** containing a chiral secondary alcohol as a second chiral element beside the biaryl axis. This should potentially lead to separable diastereomeric atropisomers [23].

**Figure:**
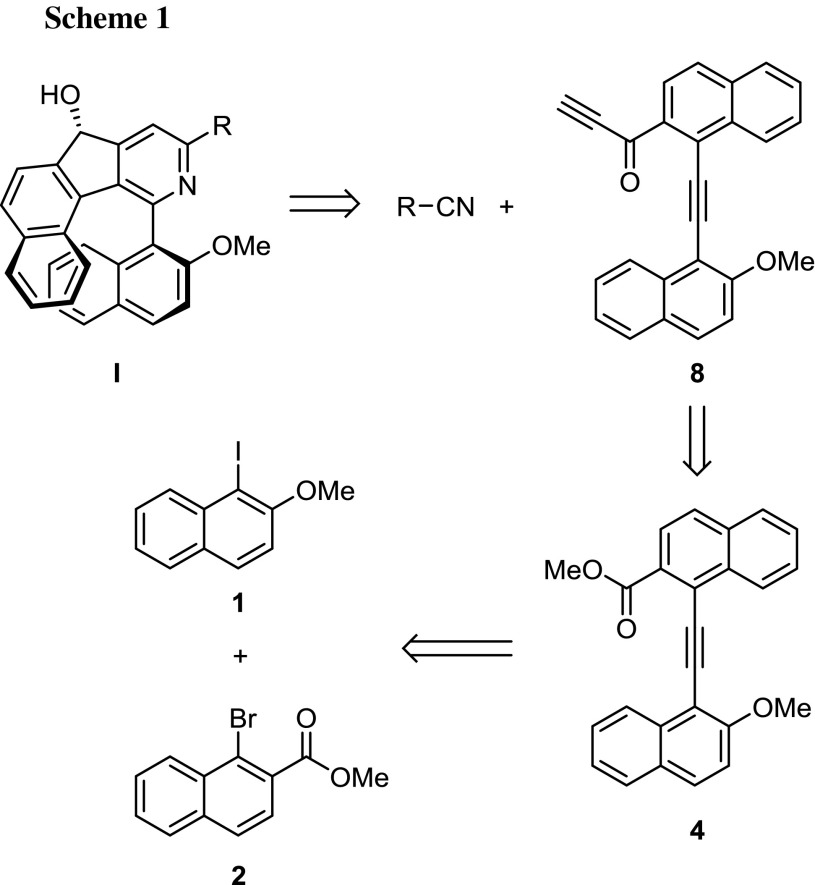

The synthesis of the building blocks **1** [24] and **2** [25] has been already described in the literature and previous work, as has the ethinylation of **1**, yielding 1-ethinyl-2-methoxynaphthalene (**3**) as the terminal alkyne [26]. Coupling of **2** and **3** was initially pursued by a copper-free cross-coupling approach reported by Stará et al. (Scheme 2) [27]. However, using piperidine or diisopropylamine (DIPA) as amine base at higher reaction temperatures required for bromides only 25 or 28% yield of **4** were obtained, in the first case together with 33% of methyl 1-(piperidin-1-yl)-2-naphthoate as byproduct from the amination of the bromonaphthalene. Instead, application of convenient Cu-/Pd-mediated Sonogashira conditions for the coupling successfully furnished target compound **4** with superior 87% yield (Scheme 2).

**Figure:**
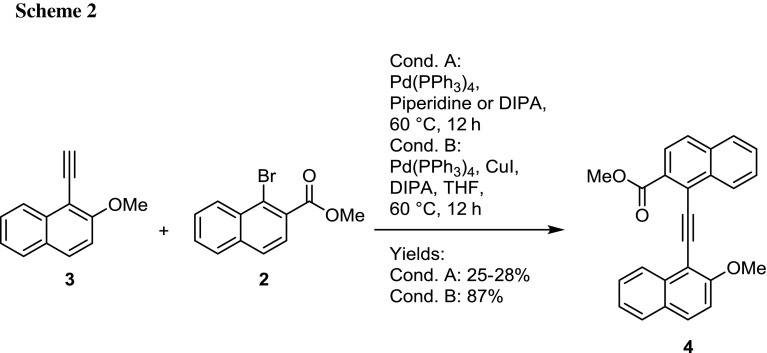

**Fig. 1:**
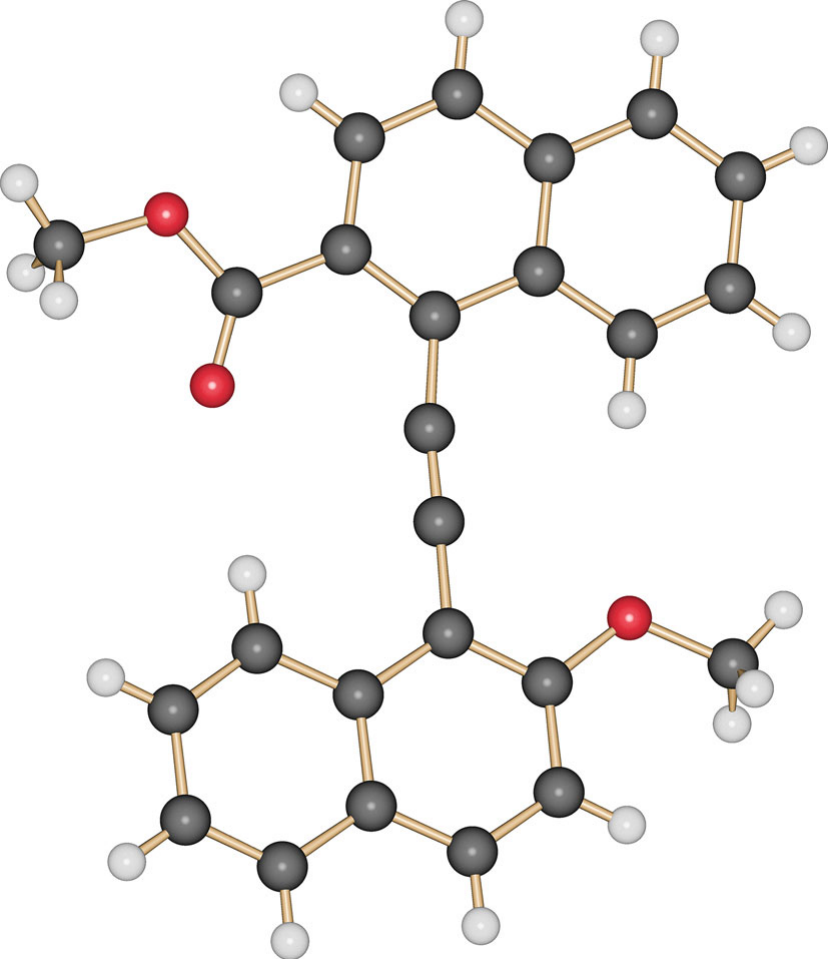
Molecular structure of compound **4** as determined by single crystal X-ray diffraction

Pure bisnaphthylalkyne **4** was isolated as yellow needles by crystallization from ethyl acetate, allowing also to gather single crystals suitable for X-ray structure analysis (Fig. 1). The substituents on the naphthyl groups point to opposite directions with the naphthyl systems being coplanar oriented to each other.

Further manipulation of **4** required the transformation of the ester moiety into a Weinreb amide for the nucleophilic addition of the second alkyne. The sequence was started by basic hydrolysis of the ester group with nearly quantitative yield of the carboxylic acid **5**. The classical approach of turning **5** into the acid chloride using thionyl chloride, followed by reaction with *N*,*O*-dimethylhydroxylamine hydrochloride (**6**) in the presence of NEt_3_ was not successful here. Fortunately application of a synthetic variation by Fu et al. gave the amide **7** in excellent yield of up to 86%, circumventing the preparation of the acid chloride (Scheme 3) [28]. The presence of triethylamine was found to be essential to promote the reaction, when using the hydrochloride, while otherwise no conversion was observed.

**Figure:**
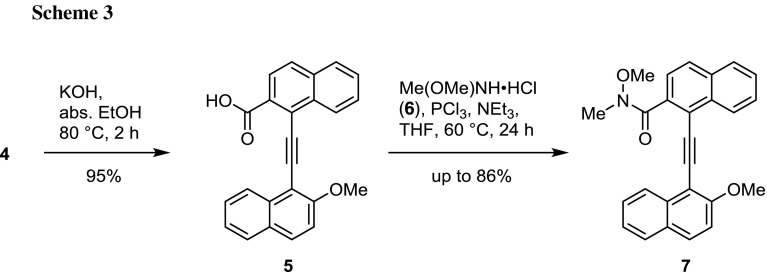

Subsequently the reaction of Weinreb amide **7** with lithiated trimethylsilylacetylene and the subsequent reduction of the synthesized ketoalkyne **8** was investigated (Scheme 4). Li-TMS-acetylide was prepared following a standard procedure and the rapid reaction with **7** was quantitative, surprisingly yielding both after work-up, the expected product **TMS-8** and the desilylated **8** with a 70:30 ratio. Subsequent desilylation of isolated **TMS-8** with KF was not met with success, as several products resulted from the reaction, possibly due to the decomposition of the activated triple bond. Prolonged reaction times of the desilylation in methanol led to double addition of MeOH to the activated triple bond. As it was planned to introduce a second element of chirality for later separation of the resulting diastereomeric atropisomers, the reduction of the ketoalkyne was pursued using (*R*)-Alpine-Borane^®^ at mild conditions. The reaction worked extremely well on both **TMS-8** and **8**, and for **TMS-8** the follow-up TMS-deprotection with KF from the crude product of the reduction proceeded as usual, allowing the isolation of enantiomerically pure **9** with 96% yield over two steps. The reduction of the keto group in isolated **8** also gave pure **9** with 96% yield.

**Figure:**
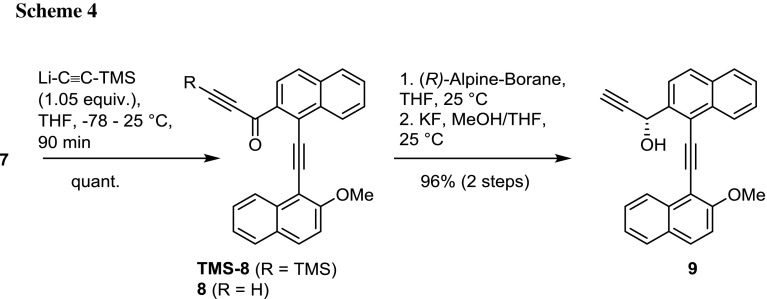

Having isolated **9** in our hands, we could study the behavior in the [2 + 2 + 2] cycloaddition reaction with different nitriles and catalysts. The first experiments utilized CpCo(COD) (CAT1) as catalyst under photochemical conditions and CpCo[P(OEt)_3_](*trans*-MeO_2_CH=CHCO_2_Me) (CAT2) under thermal conditions (Scheme 5) [21]. The latter catalyst has recently been developed in our laboratory and is commercially available [29].

**Figure:**
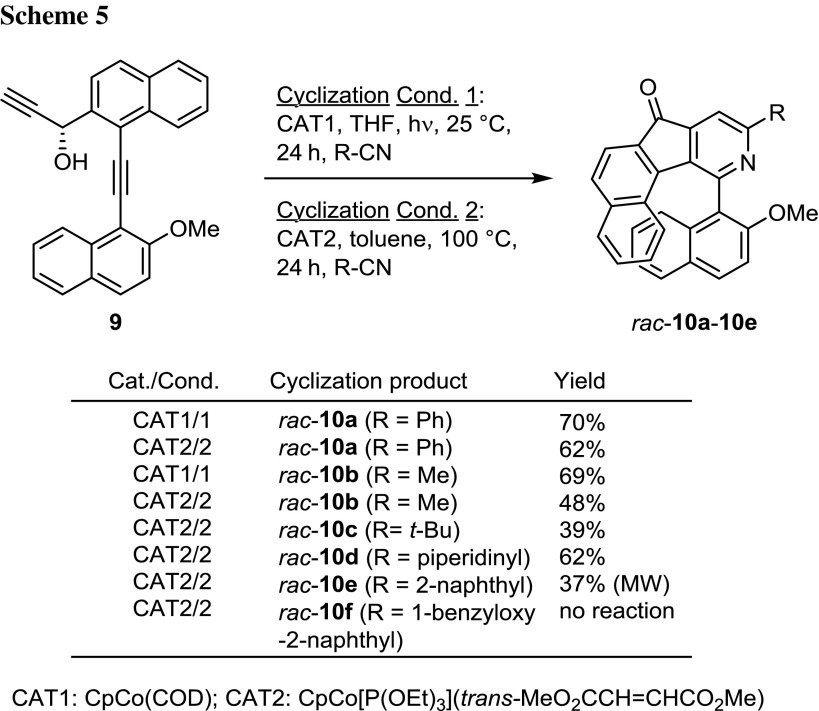

Several different nitriles were investigated and in general good yields of products were observed. A rather interesting general observation in these cyclization reactions is the dehydrogenation of the secondary alcohol group forming a keto group in the cyclization products *rac*-**10a**–**10e**, which prevents the potential isolation and separation of the intended diastereomeric atropisomers. Therefore, also reduction using an achiral hydride source such as NaBH_4_ instead of Alpine-Borane^®^ would be possible due to the later autoreoxidation. The reoxidation of the hydroxyl function might be favored by the formation of a conjugated system in the backbone of the molecule. However, the cyclization of diyne **9** under photochemical conditions proceeded very well in both cases using MeCN and PhCN as cyclization substrates, yielding up to 70% yield of racemic *rac*-**10a** and *rac*-**10b**. Yields under thermal conditions (100 °C, oil bath) using the air-stable catalyst CAT2 proceeded with slightly lower yield for *rac*-**10a** and 48% for *rac*-**10b**. No detrimental effect of the formal oxidation of the hydroxyl group on the cyclizations was identified at this point. Experiments using ketoalkyne **8** as substrate with catalysts CAT1 and CAT2 under the conditions mentioned above with PhCN only gave significantly lower yields of *rac*-**10a** (25–30%). The application of other nitriles using CAT2 under thermal conditions led to the *t*-butyl-(*rac*-**10c**) or piperidinyl-(*rac*-**10d**)-substituted biaryls in acceptable to good yields. The reaction of **9** with 2-cyanonaphthalene using CAT2 under conventional conditions gave just 17% yield of product *rac*-**10e**, while applying microwave heating (MW) to 110 °C for 1 h improved the yield of *rac*-**10e** to 37% yield. The reaction using 1-benzyloxy-2-cyanonaphthalene, however, gave no cyclization product at all under the investigated conditions.

## Conclusion

We presented the concise synthesis of a novel chiral diyne precursor molecule starting from simple arene building blocks for [2 + 2 + 2] cycloadditions. Reaction with different nitriles under thermally and photochemically assisted cobalt catalysis gave the respective annulated naphthylpyridines in good yields, while the chiral hydroxyl group was unexpectedly reoxidized to the keto group during the cyclization process. The synthesis allowed access to heterocyclic biaryl compounds possessing a stable, sterically hindered biaryl axis, which should be attractive for the synthesis of novel ligand backbones.

## Experimental

The NMR spectra were in general recorded at 298 K and the individual measurement conditions given with the data. Chemical shifts are reported in ppm relative to the ^1^H and ^13^C residue signals of the deuterated solvent (deuterochloroform: *δ* = 7.26 ppm for ^1^H and *δ* = 77.16 ppm for ^13^C). Mass spectra were obtained with a mass spectrometer at an ionizing voltage of 70 eV for EI. Only characteristic fragments containing the isotopes of highest abundance are listed. Relative intensities in percentages are given in parentheses. High-resolution mass spectroscopy (HRMS) analyses were performed using electrospray ionization/time-of-flight (ESI-TOF) mass spectrometry or electron ionization (EI) with a sector field mass analyzer. For the photochemistry two halogen lamps (460 W each) have been used for the irradiation of the thermostated Schlenk-type reaction vessel [21]. For microwave experimentation CEM Discover SP™ with glass tubes was used. All reactions were carried out in an argon atmosphere, using standard techniques in dry, oxygen-free solvents. Chromatographic purifications were done with 240–400 mesh silica gel or on an automated flash-chromatography system. CpCo(COD), Pd(PPh_3_)_4_, methyl 1-bromo-2-naphthoate (**2**) [25], and 1-ethinyl-2-methoxynaphthalene (**3**) [26] were synthesized according to known procedures. The synthesis and catalytic properties of the air-stable precatalyst CpCo[P(OEt)_3_](*trans*-MeO_2_CH=CHCO_2_Me) [CAT2]^1^ have been reported by us recently [29]. All other starting materials and compounds were commercially available and have been used as received.

### Methyl 1-[(2-methoxynaphthalen-1-yl)ethynyl]-2-naphthoate (**4**, C_25_H_18_O_3_)

*Coupling conditions A* 132.6 mg methyl 1-bromo-2-naphthoate (**2**, 0.50 mmol) together with 29 mg [Pd(PPh_3_)_4_] (0.05 mmol, 5 mol%) were dissolved in 2 cm^3^ diisopropylamine under an inert atmosphere and heated in an oil bath to 45 °C, until a clear solution resulted. Afterwards a solution of 91.1 mg 1-ethynyl-2-methoxynaphthalene (**3**, 0.50 mmol) in a mixture of diisopropylamine and THF (1 cm^3^ each) was added and the reaction mixture stirred over night at 50 °C. After cooling the solution was filtered, the filter residue washed with 5 cm^3^ toluene/petrolether (1:1 v/v) and the solvents removed in vacuo. The crude product was purified by flash column chromatography on silica gel (*n*-hexane/ethyl acetate, 100% → 66% v/v), yielding 51 mg (28% yield) pure compound. Conducting the reaction with piperidine instead of diisopropylamine gave 25% yield of **4** and in addition methyl 1-(piperidin-1-yl)-2-naphthoate (33%) as amination byproduct.

*Coupling conditions B* 764 mg compound **3** (2.88 mmol), 57.60 mg CuI (0.29 mmol, 10 mol%) and 167 mg catalyst [Pd(PPh_3_)_4_] (0.14 mmol, 5 mol%) were suspended under an inert atmosphere in 8 cm^3^ diisopropylamine and the reaction mixture heated to 45 °C, until a clear solution resulted. A second solution prepared under an inert atmosphere of 525 mg **2** (2.88 mmol) in 4 cm^3^ diisopropylamine and 8 cm^3^ THF was then added and stirred over night at 60 °C. Work-up of the crude reaction mixture like described under conditions A again using a mixture of toluene/petrolether (1:1 v/v, 50 cm^3^) for washing of the solid residuals, followed by flash column chromatography yielded the pure product (920 mg, 87% yield).

M.p.: 135–136 °C; ^1^H NMR (CDCl_3_, 300 MHz): *δ* = 4.09 (OC*H*_3_, 3H), 4.17 (COOC*H*_3_, 3H), 7.34 (d, *J* = 9.1 Hz, Ar-*H*, 1H), 7.43 (ddd, *J* = 8.2, 6.8, 1.2 Hz, Ar-*H*, 1H), 7.70 (ddd, *J* = 8.4, 6.8, 1.6 Hz, Ar-*H*, 1H), 7.60–7.68 (m, Ar-*H*, 2H), 7.81–7.92 (m, Ar-*H*, 4H), 8.05 (d, *J* = 8.7, Ar-*H*, 1H), 8.72 (d, *J* = 8.3 Hz, Ar-*H*, 1H), 9.11 (d, *J* = 8.6 Hz, Ar-*H*, 1H) ppm; ^13^C NMR (CDCl_3_, 75 MHz): *δ* = 52.6 (COO*C*H_3_), 56.8 (O*C*H_3_), 95.8 (C*C*≡CC), 96.5 (CC≡*C*C), 107.0 (Ar-*C*), 112.7 (Ar-*C*), 123.4 (Ar-*C*), 124.5 (Ar-*C*), 126.1 (2x Ar-*C*), 127.6 (Ar-*C*), 127.8 (Ar-*C*), 128.0 (Ar-*C*), 128.2 (2x Ar-*C*), 128.3 (Ar-*C*), 128.7 (Ar-*C*), 128.7 (Ar-*C*), 129.6 (Ar-*C*), 130.9 (Ar-*C*), 133.9 (Ar-*C*), 134.8 (Ar-*C*), 134.9 (Ar-*C*), 159.9 (Ar-*C*OCH_3_), 167.6 (*C*OOCH_3_) ppm; HRMS (ESI): ([C_25_H_18_O_3_] + H)^+^ calc. 367.1329, found 367.1328; ([C_25_H_18_O_3_] + Na)^+^ calc. 389.1148, found 389.1148.

CCDC-1550069 contains the supplementary crystallographic data for compound **4** (C_25_H_18_O_3_). These data can be obtained free of charge from The Cambridge Crystallographic Data Centre via http://www.ccdc.cam.ac.uk/data_request/cif.

### 1-[(2-Methoxynaphthalen-1-yl)ethynyl]-2-naphthoic acid (**5**, C_24_H_16_O_3_)

To a solution of 2.63 g KOH (46.87 mmol, 20 equiv.) in 24 cm^3^ abs. EtOH 0.86 mg of compound **4** (2.35 mmol) was added and the reaction mixture stirred for 2 h at 80 °C oil-bath temperature. The colour of the mixture changed from milky-grey to into dark purple and a solid was precipitating out of solution. For work-up the reaction mixture was diluted with 250 cm^3^ water and was washed with Et_2_O three times (200 cm^3^ each). The aqueous solution with the purple precipitate was acidified with conc. HCl (pH < 2), whereupon a colour change to yellow was observed. The suspension with the yellow solid was extracted with Et_2_O three times (200 cm^3^ each) and after desiccation of the combined phases over Na_2_SO_4_ the solvent was removed in vacuo. Pure product **5** was isolated as yellow solid (786 mg, 95%). M.p.: 179 °C (decomposition); ^1^H NMR ((CD_3_)_2_SO, 300 MHz): *δ* = 4.20 (s, OC*H*_3_, 3H), 7.43–7.51 (m, Ar-*H*, 1H), 7.57–7.66 (m, Ar-*H*, 2H), 7.71–7.78 (m, Ar-*H*, 1H), 7.82–7.89 (m, Ar-*H*, 1H), 7.93–7.99 (m, Ar-*H*, 1H), 8.02–8.12 (m, Ar-*H*, 4H), 8.72 (d, *J* = 8.4 Hz, Ar-*H*, 1H), 9.07 (d, *J* = 8.4 Hz, Ar-*H*, 1H), 13.5 (bs, O*H*) ppm; MS (EI): *m/z* (%) = 352 (100), 293 (95), 281 (28), 252 (18); HRMS (EI, 70 eV): [C_24_H_16_O_3_] calc. 352.1094, found 352.1093.

### *N*-Methoxy-1-[(2-methoxynaphthalen-1-yl)ethynyl]-*N*-methyl-2-naphthamide (**7**, C_26_H_21_NO_3_)

Optimized procedure: A solution of 1.80 g **5** (5.10 mmol) and 1.50 g *N*,*O*-dimethylhydroxylamine hydrochloride (**6**, 15.0 mmol) in 30 cm^3^ THF at 0 °C, 2.80 cm^3^ NEt_3_ (20.0 mmol), and 0.44 cm^3^ PCl_3_ (5.0 mmol) were added dropwise subsequently via syringe. The reaction mixture was stirred for 24 h at 60 °C. After 12 h additional amounts of NEt_3_ (2.80 cm^3^, 20.0 mmol) and PCl_3_ (0.44 cm^3^, 5.0 mmol) were added. After cooling the reaction mixture was quenched with sat. NaHCO_3_, extracted several time with a mixture of THF/ethyl acetate (1:1 v/v) and finally washed with brine. After drying over Na_2_SO_4_ the solvent was removed in vacuo. The crude product was further purified over silica gel by column chromatography (cyclohexane/ethyl acetate = 1:1 v/v, 1% NEt_3_). The yield obtained was 1.46 g (72%), on a smaller experimentation scale (0.5 mmol acid) 86% product were isolated. M.p.: 134–135 °C; ^1^H NMR (CDCl_3_, 300 MHz, 333 K): *δ* = 3.45 (s, NC*H*_3_, 3H), 3.64 (s, NOC*H*_3_, 3H), 4.13 (s, OC*H*_3_, 3H), 7.30 (d, *J* = 9.1 Hz, Ar-*H*, 1H), 7.41 (ddd, *J* = 7.6, 6.8, 1.3 Hz, Ar-*H*, 1H), 7.51 (d, *J* = 8.4 Hz, Ar-*H*, 1H), 7.55–7.64 (m, Ar-*H*, 2H), 7.67 (ddd, *J* = 8.3, 6.8, 1.4 Hz, Ar-*H*, 1H), 7.80 (d, *J* = 8.0 Hz, Ar-*H*, 1H), 7.83–7.92 (m, Ar-*H*, 3H), 8.48 (dd, *J* = 8.4, 1.0 Hz, Ar-*H*, 1H), 8.81 (d, *J* = 8.4 Hz, Ar-*H*, 1H) ppm; ^13^C NMR (CDCl_3_, 75 MHz): *δ* = 33.4 (N*C*H_3_), 57.1 (O*C*H_3_), 61.4 (NO*C*H_3_), 93.8 (C*C*CC), 94.7 (CC*C*C), 113.4 (Ar-*C*), 119.4 (Ar-*C*), 123.8 (Ar-*C*), 124.6 (Ar-*C*), 125.9 (Ar-*C*), 127.3 (Ar-*C*), 127.4 (Ar-*C*), 127.6 (Ar-*C*), 127.7 (Ar-*C*), 128.2 (Ar-*C*), 128.3 (Ar-*C*), 128.4 (Ar-*C*), 129.0 (Ar-*C*), 130.7 (Ar-*C*), 133.4 (Ar-*C*), 133.5 (Ar-*C*), 134.9 (Ar-*C*), 159.9 (Ar-*C*OCH_3_) ppm (not all expected signals were observed); ^13^C NMR (toluene-*d*_8_, 75 MHz, 373 K): *δ* = 33.6 (N*C*H_3_, detection of the N-Me group not possible unambiguously); MS (EI): *m/z* (%) = 364 (100), 335 (39), 276 (24), 263 (32), 132 (26); HRMS (ESI): ([C_26_H_21_NO_3_] + H)^+^ calc. 396.1594, found 396.1596; ([C_26_H_21_NO_3_] + Na)^+^ calc. 418.1414, found 418.1418; IR (ATR): $\bar{\nu }$ = 424, 466, 549, 556, 744, 753, 812, 1258, 1270, 1639 cm^−1^.

### 1-[1-[(2-Methoxynaphthalen-1-yl)ethynyl]naphthalen-2-yl]-3-(trimethylsilyl)prop-2-yn-1-one (**TMS-8**, C_29_H_24_O_2_Si) and 1-[1-[(2-methoxynaphthalen-1-yl)ethynyl]naphthalen-2-yl]prop-2-yn-1-one (**8**, C_26_H_16_O_2_)

In a secured Schlenk flask a solution of 0.19 cm^3^ trimethylsilylacetylene (1.33 mmol) in 8 cm^3^ THF was cooled to − 78 °C and 0.87 cm^3^ *n*-BuLi (1.39 mmol, 1.6 M in THF) added via syringe. The reaction mixture was allowed to warm to room temperature and stirred for further 20 min. Afterwards the solution was cooled again to − 78 °C and a solution of **7** (0.50 g, 1.26 mmol) in 8 cm^3^ THF was added dropwise. The reaction mixture was stirred for additional 90 min at room temperature after completed addition, then quenched with water and extracted several times with ethyl acetate. The combined organic phases were washed with brine, dried over Na_2_SO_4_, and the solvent removed in vacuo. Chromatographic purification at silica gel (cyclohexane/ethyl acetate 6:1 v/v) yielded compounds **TMS-8** (383 mg, 70%, yellow–orange solid) and **8** (137 mg, 30%, red–orange solid).

*Compound* ***TMS-8*** m.p.: > 119 °C (decomposition); ^1^H NMR (CDCl_3_, 300 MHz): *δ* = 0.27 (s, Si(C*H*_3_)_3_, 9H), 4.13 (s, OC*H*_3_, 3H), 7.27 (d, *J* = 9.1 Hz, Ar-*H*, 1H), 7.43 (ddd, *J* = 8.1, 6.9, 1.2 Hz, Ar-*H*, 1H), 7.60–7.69 (m, Ar-*H*, 2H), 7.74 (ddd, *J* = 8.5, 6.9, 1.3 Hz, Ar-*H*, 1H), 7.79 (d, *J* = 8.2 Hz, Ar-*H*, 1H), 7.84–7.90 (m, Ar-*H*, 3H), 8.27 (d, *J* = 8.6 Hz, Ar-*H*, 1H), 8.94 (dd, *J* = 8.6, 1.2 Hz, Ar-*H*, 1H), 9.19 (d, *J* = 8.0 Hz, Ar-*H*, 1H) ppm; ^13^C NMR (CDCl_3_, 75 MHz): *δ* = -0.6 (Si(*C*H_3_)_3_), 56.7 (O*C*H_3_), 96.2 (C*C*CC), 98.3 (CC*C*C), 100.9 (*C*CSi(CH_3_)_3_), 102.4 (*C*Si(CH_3_)_3_), 106.8 (Ar-*C*), 112.4 (Ar-*C*), 123.1 (Ar-*C*), 124.5 (Ar-*C*), 126.3 (Ar-*C*), 127.3 (Ar-*C*), 127.7 (Ar-*C*), 127.8 (Ar-*C*), 128.0 (Ar-*C*), 128.1 (Ar-*C*), 128.2 (Ar-*C*), 128.6 (Ar-*C*), 128.8 (Ar-*C*), 129.1 (Ar-*C*), 131.3 (Ar-*C*), 133.9 (Ar-*C*), 134.7 (Ar-*C*), 135.0 (Ar-*C*), 135.2 (Ar-*C*), 160.4 (Ar-*C*OCH_3_), 177.4 (*C*O) ppm; HRMS (ESI): ([C_29_H_24_O_2_Si] + H)^+^ calc. 433.1618, found 433.1617; ([C_29_H_24_O_2_Si] + Na)^+^ calc. 455.1438, found 455.1438.

*Compound* ***8*** m.p.: > 149 °C (decomposition); ^1^H NMR (CDCl_3_, 300 MHz): *δ* = 3.50 (s, CC*H*, 1H), 4.18 (s, OC*H*_3_, 3H), 7.32 (d, *J* = 9.1 Hz, Ar-*H*, 1H), 7.44 (ddd, *J* = 8.2, 6.9, 1.2 Hz, Ar-*H*, 1H), 7.63–7.75 (m, Ar-*H*, 3H), 7.81 (d, *J* = 8.1 Hz, Ar-*H*, 1H), 7.87–7.92 (m, Ar-*H*, 3H), 8.29 (d, *J* = 8.7 Hz, Ar-*H*, 1H), 8.91 (d, *J* = 8.4 Hz, Ar-*H*, 1H), 9.18–9.23 (m, Ar-*H*, 1H) ppm; HRMS (ESI): ([C_26_H_16_O_2_] + H)^+^ calc. 361.1223, found 361.1225; ([C_26_H_16_O_2_] + Na)^+^ calc. 383.1043, found 383.1043.

### (*R*)-1-[1-[(2-Methoxynaphthalen-1-yl)ethynyl]naphthalen-2-yl]prop-2-yn-1-ol (**9**, C_26_H_18_O_2_)

*Two-step procedure from* ***TMS-8***: *Reduction* A solution of 0.40 g **TMS-8** (0.93 mmol) in 20 cm^3^ THF was reacted under inert conditions with 3.30 cm^3^ (*R*)-Alpine-Borane^®^ (1.17 mmol, 0.5 M in THF) for 6 days at room temperature. Acetaldehyde (0.10 cm^3^, 1.8 mmol) was added to quench the reaction and the mixture heated to 40 °C for 1 h. After removal of volatile components in vacuo the oily residue was dissolved in Et_2_O, the solution cooled to 0 °C and 0.25 cm^3^ ethanolamine (4.17 mmol) added. After 20 min ethyl acetate was added and the resulting organic phase washed with water and brine. The phase was dried over Na_2_SO_4_ and the solvent evaporated.

*Deprotection* The residue was directly subjected to deprotection and, therefore, dissolved in a mixture of THF and methanol (15 cm^3^ each) and 484 mg KF (8.33 mmol) added as solid. The reaction mixture was stirred at room temperature for 48 h until TLC control indicated complete consumption of the starting material. The mixture was diluted with 30 cm^3^ water and extracted several times with ethyl acetate. The combined organic phases were washed with water and brine and dried over Na_2_SO_4_. Purification by chromatography on silica gel (cyclohexane:ethyl acetate 6:1 v/v) gave the pure product **9** (321 mg, 96% yield over two steps).

*Reduction from compound* ***8*** A solution of 0.17 g **8** (0.47 mmol) in 12 cm^3^ THF was mixed under inert conditions with 1.70 cm^3^ (*R*)-Alpine-Borane^®^ (0.85 mmol, 0.5 M in THF) and allowed to react for 6 days at room temperature. Acetaldehyde (0.05 cm^3^, 0.9 mmol) was added to quench the reaction and the mixture heated to 40 °C for 1 h. After the removal of volatile components in vacuo the oily residue was dissolved in 6 cm^3^ Et_2_O, the solution cooled to 0 °C and 0.12 cm^3^ ethanolamine (1.80 mmol) added. After 20 min ethyl acetate was added and the resulting organic phase washed with water and brine. The phase was dried over Na_2_SO_4_ and the solvent evaporated. The residue was purified by chromatography on silica gel (cyclohexane/ethyl acetate 6:1 v/v) to give pure product **9** (166 mg, 96% yield).

$[\alpha ]_{\text{D}}^{23}$ = − 25.0° (*c* = 0.934, CHCl_3_); ^1^H NMR (CDCl_3_, 300 MHz): *δ* = 2.72 (d, *J* = 2.3 Hz, C*H*, 1H), 4.15 (s, OC*H*_3_, 3H), 4.08 (bs, HCO*H*, 1H), 6.33 (bs, *H*COH, 1H), 7.34 (d, *J* = 9.2 Hz, Ar-*H*, 1H), 7.45 (ddd, *J* = 8.2, 6.9, 1.2 Hz, Ar-*H*, 1H), 7.57 (ddd, *J* = 8.1, 6.8, 1.3 Hz, Ar-*H*, 1H), 7.63–7.71 (m, Ar-*H*, 2H), 7.85 (d, *J* = 8.1 Hz, Ar-*H*, 1H), 7.87–7.93 (m, Ar-*H*, 4H), 8.49 (d, *J* = 8.4 Hz, Ar-*H*, 1H), 8.70 (d, *J* = 8.4 Hz, Ar-*H*, 1H) ppm; ^13^C NMR (CDCl_3_, 75 MHz): *δ* = 56.7 (O*C*H_3_), 64.0 (H*C*OH), 75.2 (*C*CH), 83.0 (C*C*H), 94.8 (Ar-CC*C*C-Ar), 95.9 (Ar-C*C*CC-Ar), 106.3 (Ar-*C*), 112.4 (Ar-*C*), 119.8 (Ar-*C*), 124.6 (2x Ar-*C*), 125.4 (Ar-*C*), 126.8 (Ar-*C*), 126.9 (Ar-*C*), 127.4 (Ar-*C*), 128.0 (Ar-*C*), 128.4 (2x Ar-*C*), 128.7 (Ar-*C*), 129.0 (Ar-*C*), 130.9 (Ar-*C*), 133.2 (Ar-*C*), 133.3 (Ar-*C*), 134.1 (Ar-*C*), 140.7 (Ar-*C*), 159.6 (*C*OCH_3_) ppm; HRMS (ESI): ([C_26_H_18_O_2_] + Na)^+^ calc. 385.1199, found 383.1200.

### General procedures for cyclization reactions under thermal and photochemical conditions

*Cyclization cond. A* A thermostated photochemical reactor was loaded with catalyst CAT1 under inert conditions and a solution of diyne **8** or **9** in 15 cm^3^ THF was added, followed by the appropriate nitrile. The reaction mixture was irradiated for 24 h at 25 °C using medium-pressure metal halide lamps (2 × 450 W). For stopping the reaction the lamps were turned off and the reaction vessel opened to air. The reaction solution was filtered over Celite and the solvent was removed in vacuo. The crude product was purified by automated flash chromatography (cyclohexane/ethyl acetate, 100% → 66% cyclohexane), yielding the pure product.

*Cyclization cond. B* In a Schlenk flask catalyst CAT2 was weighted under inert conditions, followed by addition of a solution of diyne **8** or **9** in 3 cm^3^ toluene and the appropriate nitrile. The reaction mixture was heated to 100 °C for 24 h. After cooling the reaction solution was filtered over Celite and the solvent was removed in vacuo. The crude product was purified by automated flash chromatography (cyclohexane/ethyl acetate, 100% → 66% cyclohexane), yielding the pure product.

### (*rac*)-11-(2-Methoxynaphthalen-1-yl)-9-phenyl-7*H*-benzo[6,7]indeno[1,2-c]pyridin-7-one (**10a**, C_33_H_21_O_2_)

*Syntheses following cyclization cond. A* Reacting 60 mg **8** (0.17 mmol) with 3.88 mg CAT1 (16.7 µmol) and 85 mm^3^ PhCN (0.83 mmol) gave biaryl **10a** with 25% (19 mg) yield. Reacting 78 mg **9** (0.22 mmol) with 5.0 mg CAT1 (21.5 µmol) and 110 mm^3^ PhCN (1.08 mmol) gave biaryl **10a** with 70% (70 mg) yield.

*Syntheses following cyclization cond. B* Reacting 60 mg **8** (0.17 mmol) with 7.8 mg CAT2 (18 µmol) and 85 mm^3^ PhCN (0.83 mmol) gave biaryl **10a** with 30% (23 mg) yield. Reacting 100 mg **9** (0.28 mmol) with 12.8 mg CAT2 (29.5 µmol) and 140 mm^3^ PhCN (1.38 mmol) gave biaryl **10a** with 62% (80 mg) yield.

M.p.: 99–100 °C; ^1^H NMR (CDCl_3_, 300 MHz): *δ* = 3.25 (s, OC*H*_3_, 3H), 6.59 (ddd, *J* = 8.7, 6.9, 1.3 Hz, Ar-*H*, 1H), 6.85 (d, *J* = 8.6 Hz, Ar-*H*, 1H), 7.16 (d, *J* = 9.1 Hz, Ar-*H*, 1H), 7.29 (ddd, *J* = 8.3. 6.8, 1.1 Hz, Ar-*H*, 1H), 7.41–7.54 (m, Ar-*H*, 5H), 7.69–7.85 (m, Ar-*H*, 3H), 7.92–7.97 (m, Ar-*H*, 1H), 8.02 (d, *J* = 9.1 Hz, Ar-*H*, 1H), 8.10 (s, Ar-*H*, 1H), 8.12–8.17 (m, Ar-*H*, 3H) ppm; ^13^C NMR (CDCl_3_, 75 MHz): *δ* = 56.1 (O*C*H_3_), 112.0 (Ar-*C*), 113.5 (Ar-*C*), 119.8 (Ar-*C*), 124.3 (Ar-*C*), 124.6 (Ar-*C*), 125.6 (Ar-*C*), 126.3 (Ar-*C*), 126.5 (Ar-*C*), 127.1 (2x Ph-*C*), 127.5 (Ar-*C*), 128.1 (Ar-*C*), 128.6 (Ar-*C*), 128.9 (2x Ar-*C*), 129.7 (Ar-*C*), 129.8 (Ar-*C*), 130.6 (Ar-*C*), 131.2 (Ar-*C*), 132.5 (Ar-*C*), 133.3 (Ar-*C*), 138.4 (2x Ar-*C*), 138.7 (Ar-*C*), 144.0 (Ar-*C*), 146.9 (Ar-*C*), 149.3 (Ar-*C*), 155.1 (Ar-*C*), 158.3 (Ar-*C*), 194.0 (*CO*) ppm; HRMS (ESI): ([C_33_H_21_NO_2_] + H)^+^ calc. 464.1645, found 464.1646; ([C_33_H_21_NO_2_] + Na)^+^ calc. 468.1465, found 468.1459; UV–Vis (THF, *c* ≈ 10^−3^): *λ*_max_ = 245, 285, 294, 299, 468 (broad signal) nm; IR (ATR): $\bar{\nu }$ = 692, 747, 727, 810, 1072, 1246, 1260, 1367, 1381 1711 cm^−1^.

### (*rac*)-11-(2-Methoxynaphthalen-1-yl)-9-methyl-7*H*-benzo[6,7]indeno[1,2-c]pyridin-7-one (**10b**, C_28_H_19_NO_2_)

*Syntheses following cyclization cond. A* Reacting 124 mg **9** (0.34 mmol) with 7.9 mg CAT1 (34 µmol) and 90 mm^3^ MeCN (1.71 mmol) gave biaryl **10b** with 69% (95 mg) yield.

*Syntheses following cyclization cond. B* Reacting 100 mg **9** (0.27 mmol) with 12.8 mg CAT2 (29.5 µmol) and 73 mm^3^ MeCN (1.38 mmol) gave biaryl **10b** with 48% (53 mg) yield.

M.p.: > 224 °C (decomposition); ^1^H NMR (CDCl_3_, 300 MHz): *δ* = 2.72 (s, CC*H*_3_, 3H), 3.33 (s, OC*H*_3_, 3H), 6.54 (ddd, *J* = 8.9, 6.8, 1.3 Hz, Ar-*H*, 1H), 6.76 (d, *J* = 8.6 Hz, Ar-*H*, 1H), 7.19 (d, *J* = 9.1 Hz, Ar-*H*, 1H), 7.25 (ddd, *J* = 8.2, 6.8, 1.1 Hz, Ar-*H*, 1H), 7.36–7.49 (m, Ar-*H*, 3H), 7.65–7.81 (m, Ar-*H*, 3H), 7.84–7.93 (m, Ar-*H*, 2H), 8.0 (d, *J* = 9.0 Hz, Ar-*H*, 1H) ppm; ^13^C NMR (CDCl_3_, 75 MHz): *δ* = 25.0 (C*C*H_3_), 56.2 (O*C*H_3_), 113.6 (Ar-*C*), 115.6 (Ar-*C*), 119.7 (Ar-*C*), 124.3 (Ar-*C*), 124.5 (Ar-*C*), 125.2 (Ar-*C*), 126.3 (Ar-*C*), 126.5 (Ar-*C*), 127.5 (Ar-*C*), 128.1 (Ar-*C*), 128.3 (Ar-*C*), 128.5 (Ar-*C*), 128.9 (Ar-*C*), 129.7 (Ar-*C*), 130.5 (Ar-*C*), 131.1 (Ar-*C*), 132.2 (Ar-*C*), 133.2 (Ar-*C*), 137.5 (Ar-*C*), 138.4 (Ar-*C*), 143.3 (Ar-*C*), 146.9 (Ar-*C*), 149.0 (Ar-*C*), 155.0 (Ar-*C*), 160.7 (Ar-*C*), 194.3 (*CO*) ppm; HRMS (ESI): ([C_28_H_19_NO_2_] + H)^+^ calc. 402.1489, found 402.1491; ([C_28_H_19_NO_2_] + Na)^+^ calc. 424.1308, found 424.1308; IR (ATR): $\bar{\nu }$ = 436, 735, 748, 760, 780, 797, 807, 1244, 1257, 1713 cm^−1^.

### (*rac*)-11-(2-Methoxynaphthalen-1-yl)-9-tert-butyl-7*H*-benzo[6, 7]indeno-[1,2-c]pyridin-7-one (**10c**, C_31_H_25_NO_2_)

Syntheses following cyclization cond. B: the reaction of 40 mg diyne **9** (0.11 mmol), 5.1 mg CAT2 (0.011 mmol), and 28 mg *tert*-butylnitrile (0.33 mmol) furnished biaryl **10c** with 39% (19 mg) yield.

^1^H NMR (CDCl_3_, 300 MHz): *δ* = 1.43 (s, C(C*H*_3_)_3_, 9H), 3.18 (s, OC*H*_3_, 3H), 6.55 (ddd, *J* = 8.8, 6.8, 1.4 Hz, Ar-*H*, 1H), 6.77 (dd, *J* = 8.7, 1.1 Hz, Ar-*H*, 1H), 7.12 (d, *J* = 9.1 Hz, Ar-*H*, 1H), 7.25 (ddd, *J* = 8.2, 6.8, 1.1 Hz, Ar-*H*, 1H), 7.38–7.48 (m, Ar-*H*, 2H), 7.65 (s, Ar-*H*, 1H), 7.66–7.73 (m, Ar-*H*, 2H), 7.78 (d, *J* = 8.2 Hz, Ar-*H*, 1H), 7.88–7.93 (m, Ar-*H*, 1H), 7.97 (d, *J* = 9.1 Hz, Ar-*H*, 1H), 8.04–8.08 (m, Ar-*H*, 1H) ppm; ^13^C NMR (CDCl_3_, 75 MHz): *δ* = 30.1 (C(*C*H_3_)_3_), 38.4 (*C*(CH_3_)_3_), 56.2 (O*C*H_3_), 111.2 (Ar-*C*), 113.9 (Ar-*C*), 119.7 (Ar-*C*), 124.2 (Ar-*C*), 125.9 (Ar-*C*), 126.2 (Ar-*C*), 126.5 (Ar-*C*), 127.1 (Ar-*C*), 128.0 (Ar-*C*), 128.2 (Ar-*C*), 128.4 (Ar-*C*), 128.8 (Ar-*C*), 129.7 (Ar-*C*), 130.2 (Ar-*C*), 131.0 (Ar-*C*), 132.4 (Ar-*C*), 133.3 (Ar-*C*), 138.3 (Ar-*C*), 143.4 (Ar-*C*), 147.9 (Ar-*C*), 155.0 (Ar-*C*), 160.9 (Ar-*C*), 171.2 (Ar-*C*), 194.7 (*CO*) ppm (not all expected signals were found, possibly due to signal overlap); HRMS (ESI): ([C_31_H_25_NO_2_])^+^ calc. 444.1958, found 444.1951.

### (*rac*)-11-(2-Methoxynaphthalen-1-yl)-9-piperidinyl-7*H*-benzo[6,7]indeno-[1,2-c]pyridin-7-one (**10d**, C_32_H_26_N_2_O_2_)

*Syntheses following cyclization cond. B* Reacting 40 mg **9** (0.11 mmol) with 5.1 mg CAT2 (0.011 mmol) and 40 mm^3^ 1-piperidinecarbonitrile (0.33 mmol) gave biaryl **10d** with 62% yield (32 mg).

^1^H NMR (CDCl_3_, 300 MHz): *δ* = 1.61–1.72 (m, C_5_H_10_N-*H*, 6H), 3.30 (s, OC*H*_3_, 3H), 3.66–3.75 (m, C_5_H_10_N-*H*, 4H), 6.50 (ddd, *J* = 8.7, 6.7, 1.3 Hz, Ar-*H*, 1H), 6.73 (dd, *J* = 8.8, 0.9 Hz, Ar-*H*, 1H), 7.13 (s, Ar-*H*, 1H), 7.16 (d, *J* = 9.1 Hz, Ar-*H*, 1H), 7.22 (ddd, *J* = 8.3, 6.8, 1.2 Hz, Ar-*H*, 1H), 7.36–7.46 (m, Ar-*H*, 2H), 7.57 (d, *J* = 8.2 Hz, Ar-*H*, 1H), 7.63 (d, *J* = 8.2 Hz, Ar-*H*, 1H), 7.73 (d, *J* = 8.3 Hz, Ar-*H*, 1H), 7.87–7.91 (m, Ar-*H*, 1H), 7.96 (d, *J* = 9.1 Hz, Ar-*H*, 1H), 8.08–8.11 (m, Ar-*H*, 1H) ppm; ^13^C NMR (CDCl_3_, 75 MHz): *δ* = 24.8 (C_5_H_10_N-*C*), 25.7 (2x C_5_H_10_N-*C*), 46.3 (2x C_5_H_10_N-*C*), 56.2 (O*C*H_3_), 100.8 (Ar-*C*), 113.8 (Ar-*C*), 119.7 (Ar-*C*), 123.9 (Ar-*C*), 125.5 (Ar-*C*), 125.8 (Ar-*C*), 126.7 (Ar-*C*), 126.8 (Ar-*C*), 126.9 (Ar-*C*), 127.7 (Ar-*C*), 128.0 (2x Ar-*C*), 128.2 (Ar-*C*), 128.5 (Ar-*C*), 129.4 (Ar-*C*), 130.4 (Ar-*C*), 131.5 (Ar-*C*), 133.3 (Ar-*C*), 138.3 (Ar-*C*), 145.6 (Ar-*C*), 148.8 (Ar-*C*), 154.7 (Ar-*C*), 158.6 (Ar-*C*), 194.6 (*CO*) ppm (not all expected signals could be observed individually); HRMS (ESI): ([C_32_H_26_N_2_O_2_] + H)^+^ calc. 471.2067, found 471.2059.

### (*rac*)-11-(2-Methoxynaphthalen-1-yl)-9-(naphthalen-1-yl)-7*H*-benzo[6,7]-indeno[1,2-c]pyridin-7-one (**10e**, C_37_H_23_NO_2_)

Syntheses following cyclization cond. B: the reaction of 50 mg diyne **9** (0.137 mmol), 6.4 mg CAT2 (0.0137 mmol), and 42.3 mg 1-naphthonitrile (0.276 mmol) under the original conditions of cond. B gave biaryl **10b** with 17% yield (12 mg).

Alternative syntheses following cyclization cond. B (instead of conventional heating microwave heating (MW) was applied: 1 h, 110 °C, 200 W): A solution of 40 mg diyne **9** (0.11 mmol) with 5.1 mg CAT2 (0.011 mmol), and 34 mg 1-naphthonitrile (0.22 mmol) in 3 cm^3^ toluene yielded upon microwave irradiation 21 mg of biaryl **10e** (37% yield).

^1^H NMR (CDCl_3_, 300 MHz): *δ* = 3.50 (s, OC*H*_3_, 3H), 6.60 (ddd, *J* = 8.7, 6.8, 1.4 Hz, Ar-*H*, 1H), 6.94 (dd, *J* = 8.7, 1.0 Hz, Ar-*H*, 1H), 7.23–7.31 (m, Ar-*H*, 2H), 7.37 (ddd, *J* = 8.0, 6.9, 1.2 Hz, Ar-*H*, 1H), 7.44 (ddd, *J* = 8.5, 6.8, 1.4 Hz, Ar-*H*, 1H), 7.49–7.59 (m, Ar-*H*, 3H), 7.71 (d, *J* = 8.2 Hz, Ar-*H*, 1H), 7.79 (d, *J* = 8.1 Hz, Ar-*H*, 1H), 7.82 (dd, *J* = 7.2, 1.3 Hz, Ar-*H*, 1H), 7.84–7.93 (m, Ar-*H*, 4H), 7.97–8.03 (m, Ar-*H*, 3H),8.41–8.45 (m, Ar-*H*, 1H) ppm; ^13^C NMR (CDCl_3_, 75 MHz): *δ* = 56.3 (O*C*H_3_), 113.5 (Ar-*C*), 117.0 (Ar-*C*), 119.8 (Ar-*C*), 124.2 (Ar-*C*), 125.2 (Ar-*C*), 125.5 (Ar-*C*), 125.9 (Ar-*C*), 126.1 (Ar-*C*), 126.5 (Ar-*C*), 126.6 (Ar-*C*), 126.8 (Ar-*C*), 127.5 (Ar-*C*), 127.8 (Ar-*C*), 128.3 (2x Ar-*C*), 128.6 (2x Ar-*C*), 129.0 (Ar-*C*), 129.5 (Ar-*C*), 129.7 (Ar-*C*), 130.9 (Ar-*C*), 131.2 (2x Ar-*C*), 132.5 (Ar-*C*), 133.3 (Ar-*C*), 134.2 (Ar-*C*), 138.4 (Ar-*C*), 138.6 (Ar-*C*), 143.4 (Ar-*C*), 146.5 (Ar-*C*), 149.5 (Ar-*C*), 155.1 (Ar-*C*), 160.9 (Ar-*C*), 194.0 (*CO*) ppm (not all expected signals could be observed individually); HRMS (ESI): ([C_37_H_23_NO_2_] + H)^+^ calc. 514.1802, found 514.1795.

## Electronic supplementary material

Below is the link to the electronic supplementary material.

Supplementary material 1 (DOCX 2996 kb)

## Footnotes

1 Commercially available from TCI, order no. C3165, CAS no. 1487195-87-8 (http://www.tcichemicals.com/).
