# Supplementary material for: Novel naphthylpyridines from cobalt-catalyzed cyclotrimerization of a chiral diyne
Source: Monatsh Chem. 2017 Nov 28;149(4):755–63. doi: 10.1007/s00706-017-2083-9 (PMC5906485; doi:10.1007/s00706-017-2083-9)
Supplement: Supplementary file 1 — Supplementary material 1 (DOCX 2996 kb) [file 706_2017_2083_MOESM1_ESM.docx]

Novel naphthylpyridines from cobalt-catalyzed cyclotrimerization of a chiral diyne

Volkmar Trommer^[1]^, Fabian Fischer^[1]^, Marko Hapke*^[1,2]^

^[1]^ Leibniz-Institut für Katalyse e.V. an der Universität Rostock (LIKAT Rostock), Albert-Einstein-Str. 29a, D-18059 Rostock (Germany)

^[2]^ Institut für Katalyse (INCA), Johannes Kepler Universität Linz, Altenberger Str. 69, A-4040 Linz (Austria)

**Table of Contents**

[1. General Methods 2](#_Toc493002031)

[2. NMR Spectra 3](#_Toc493002032)

# 1. General Methods

All experiments were carried out under inert gas atmosphere (argon) in flame-dried Schlenk glassware. The anhydrous solvents (tetrahydrofuran, toluene, dichloromethane and *n*-hexane) were dried in a solvent purification system MD-5 from Inert (former Innovative Technology) or by standard purification and drying methods. All NMR spectra were recorded on either a Bruker AV 300, AV 400 or Fourier 300 NMR spectrometer. HRMS (ESI-TOF) was performed with a Agilent 6210 Time-of-Flight LC/MS and HRMS (EI) and MS (EI) on a Thermo Electron Finnigan MAT 95-XP or an Agilent 6890 N/5973. Optical rotation was measured on an Anton Paar polarimeter MCP 200 and melting points with a Mettler Toledo MP70 melting point system. X-Ray analysis was performed on a Bruker Kappa APEX II Duo diffractometer.

# 2. NMR Spectra

**Compound 4**, ^1^H NMR (CDCl_3_)

**
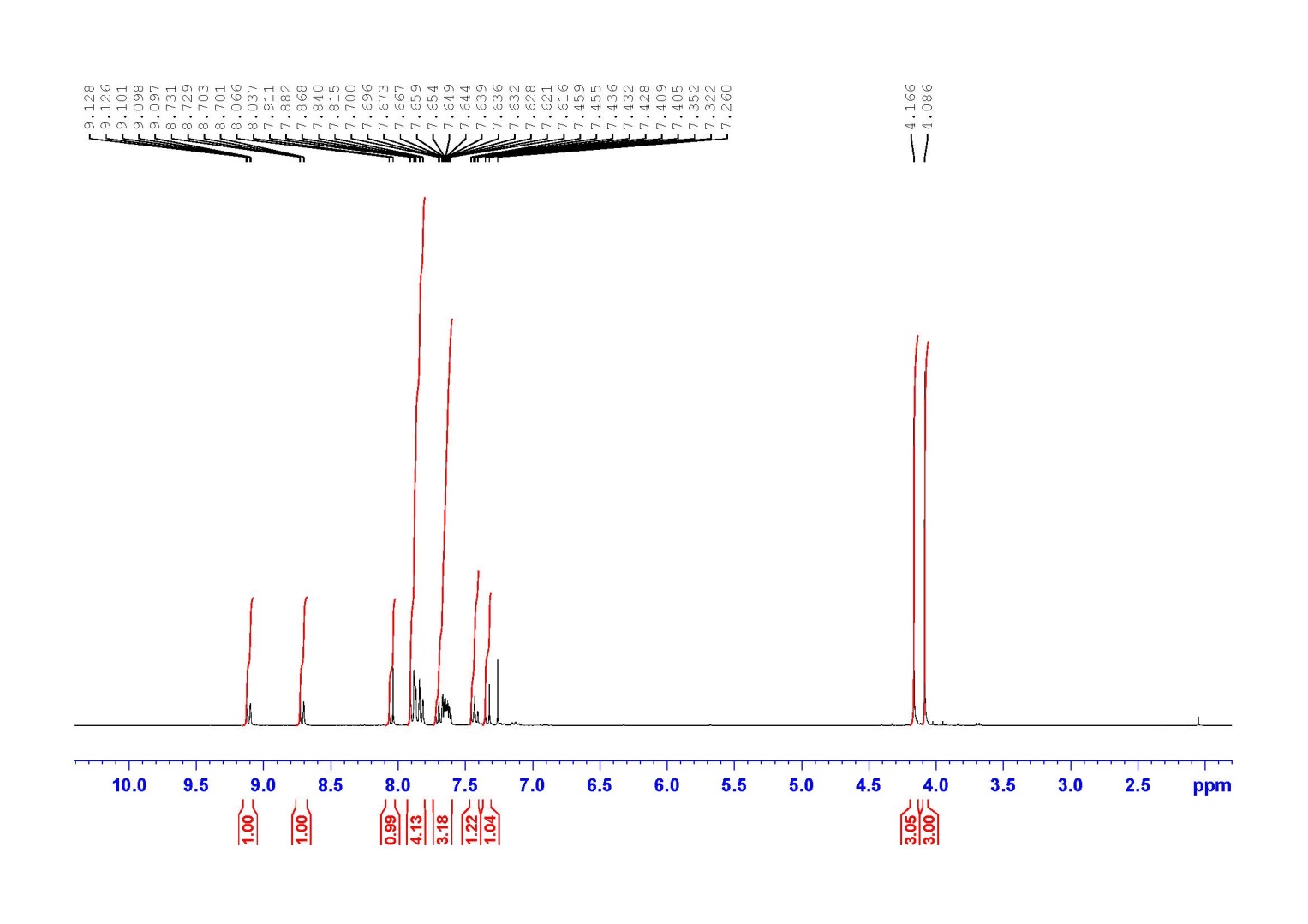
**

^13^C NMR (CDCl_3_)

**
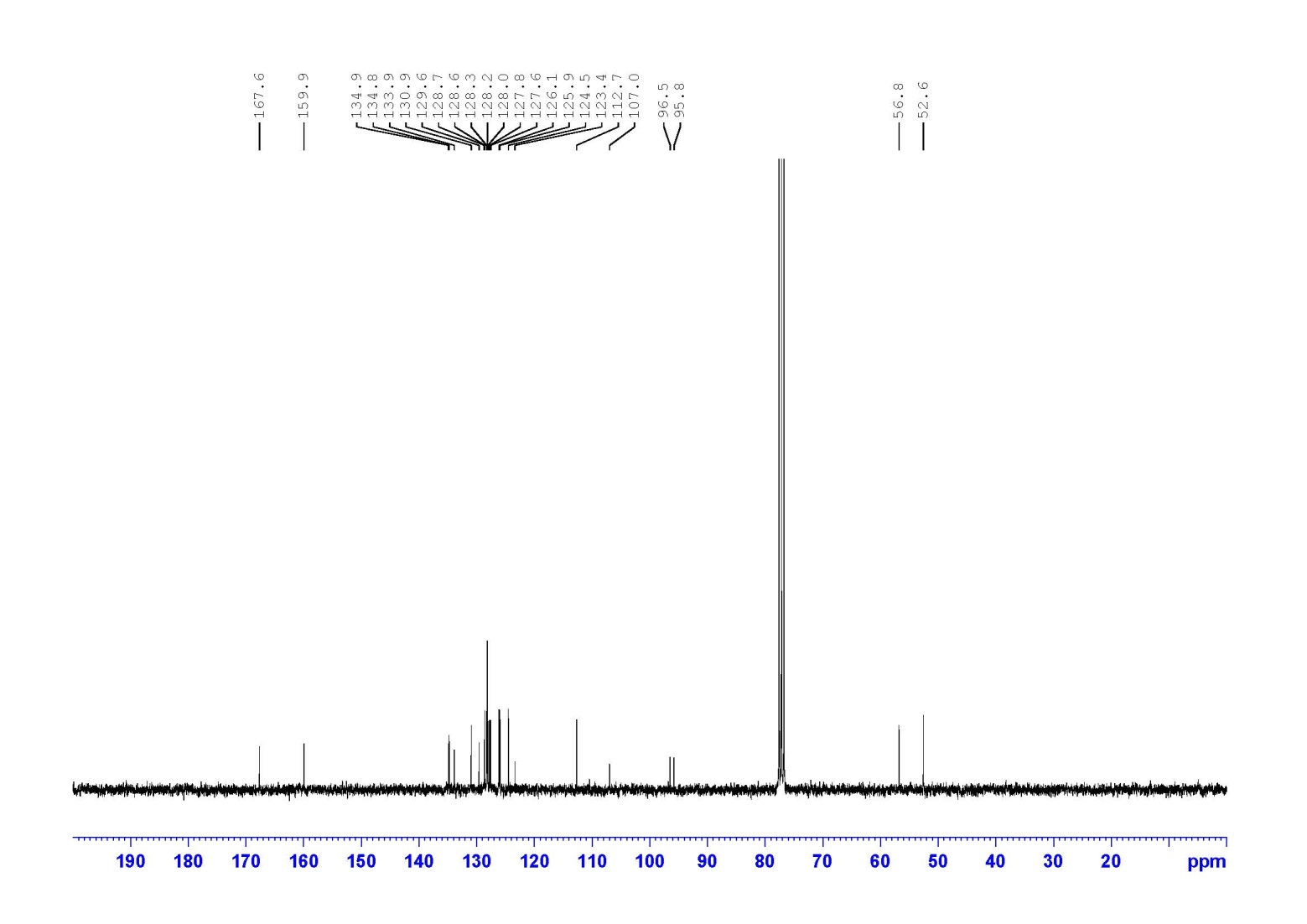
**

**Compound 5**, ^1^H NMR (DMSO-d_6_)


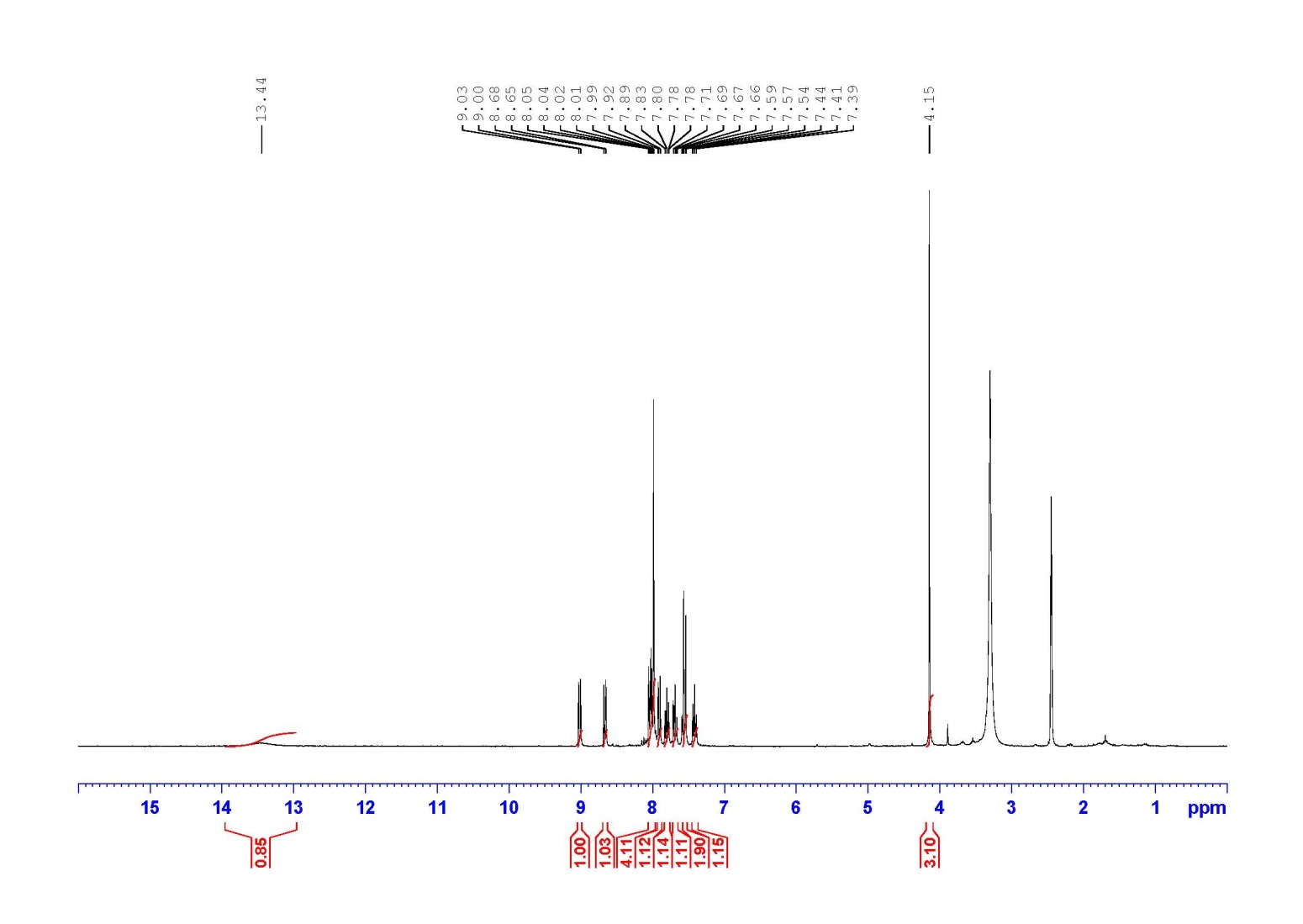

H_2_O

residual DMSO

**Compound 7**, ^1^H NMR (CDCl_3_)


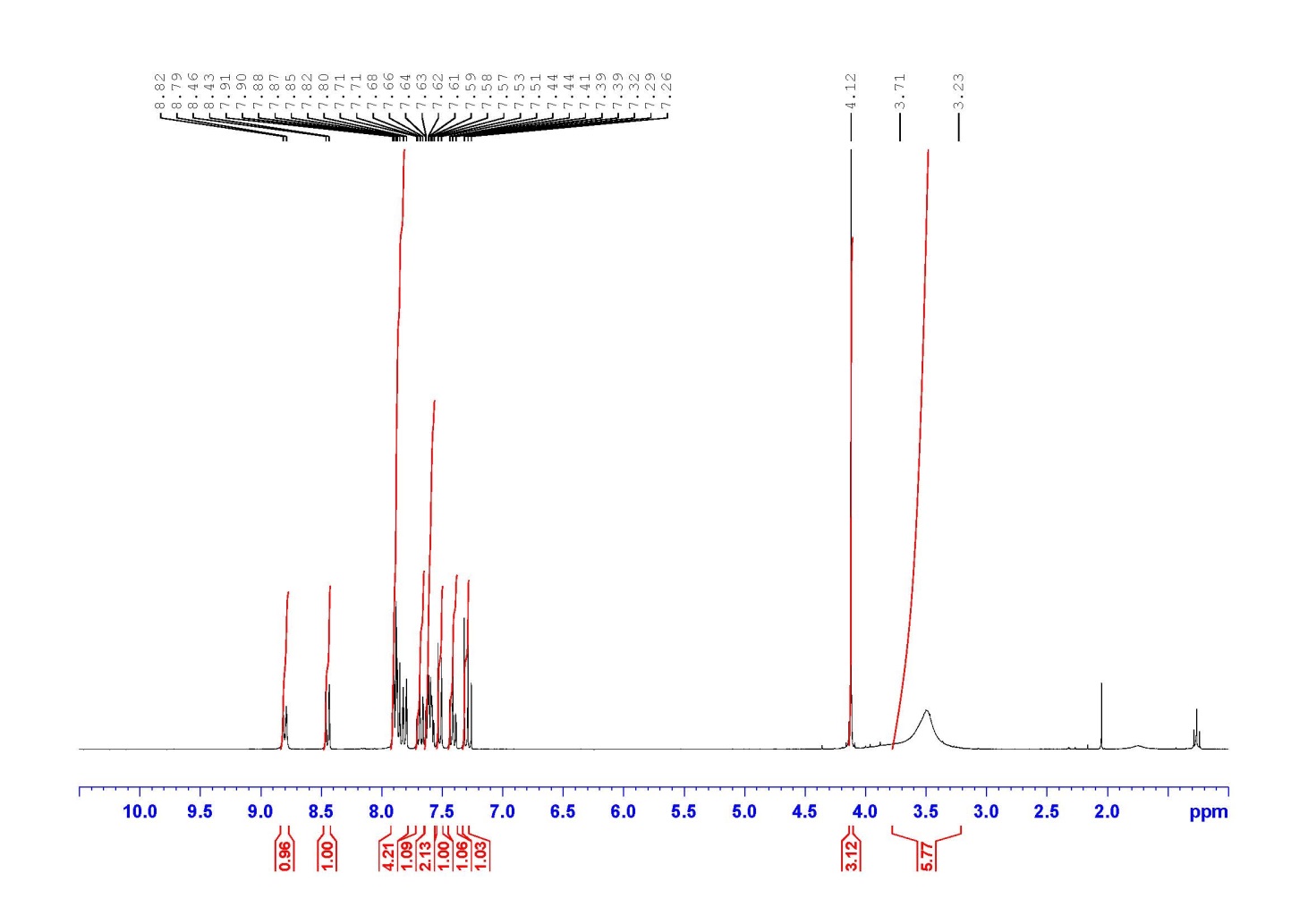

^13^C NMR (CDCl_3_)


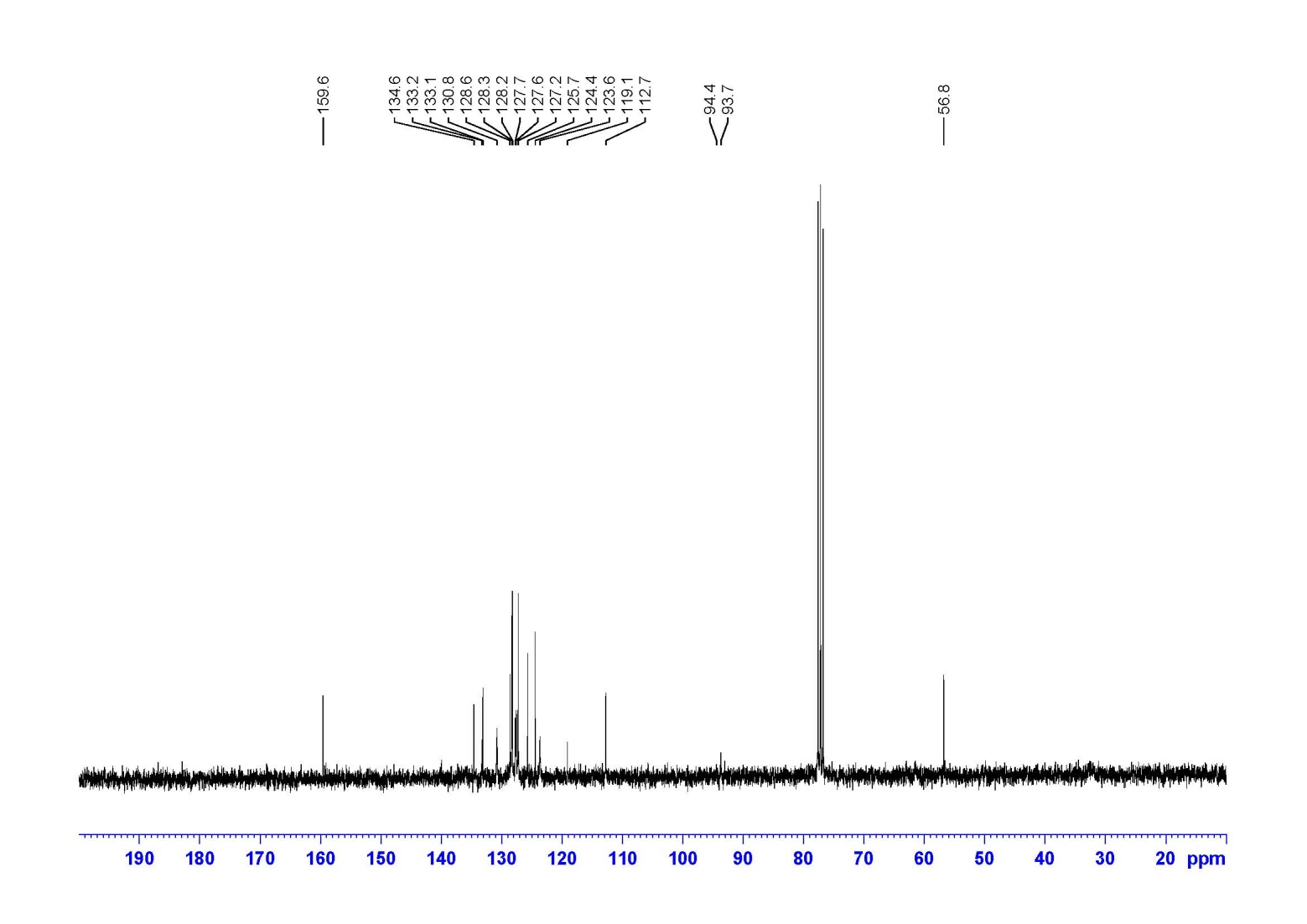


**Compound 7**, ^1^H NMR (CDCl_3_) *at 333K*


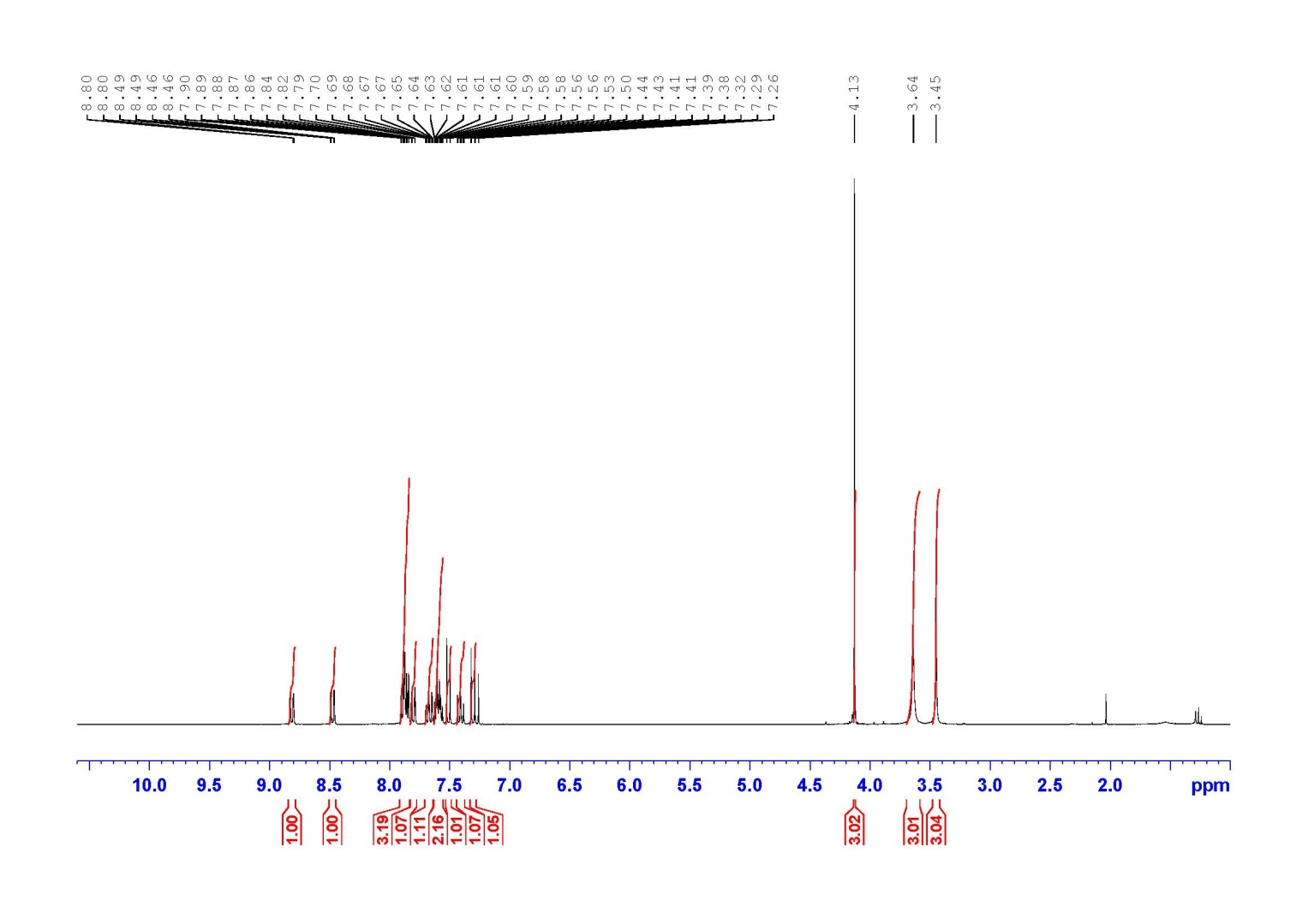

^13^C NMR (CDCl_3_) *at 333K*


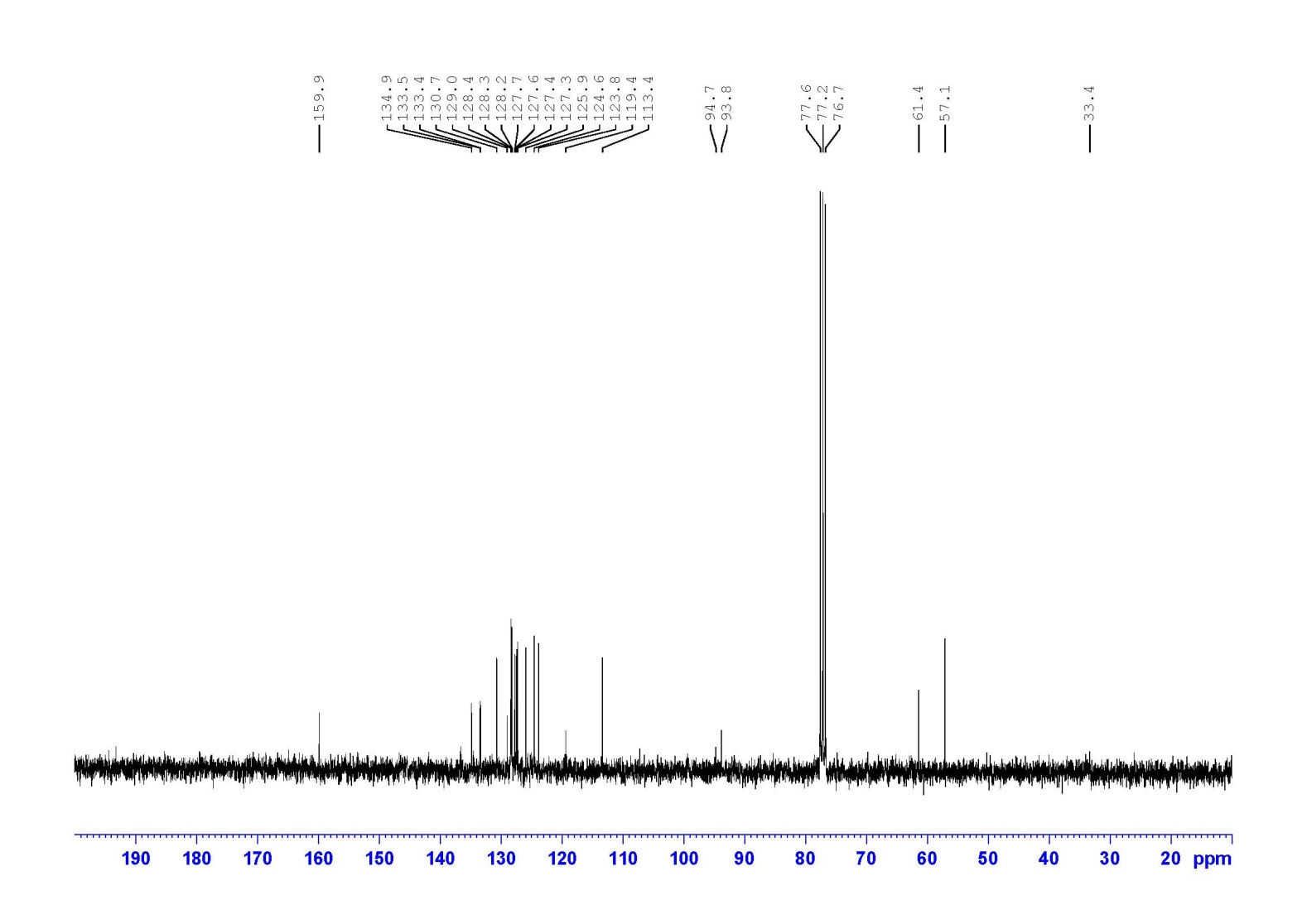


**Compound 8**, ^1^H NMR (CDCl_3_)


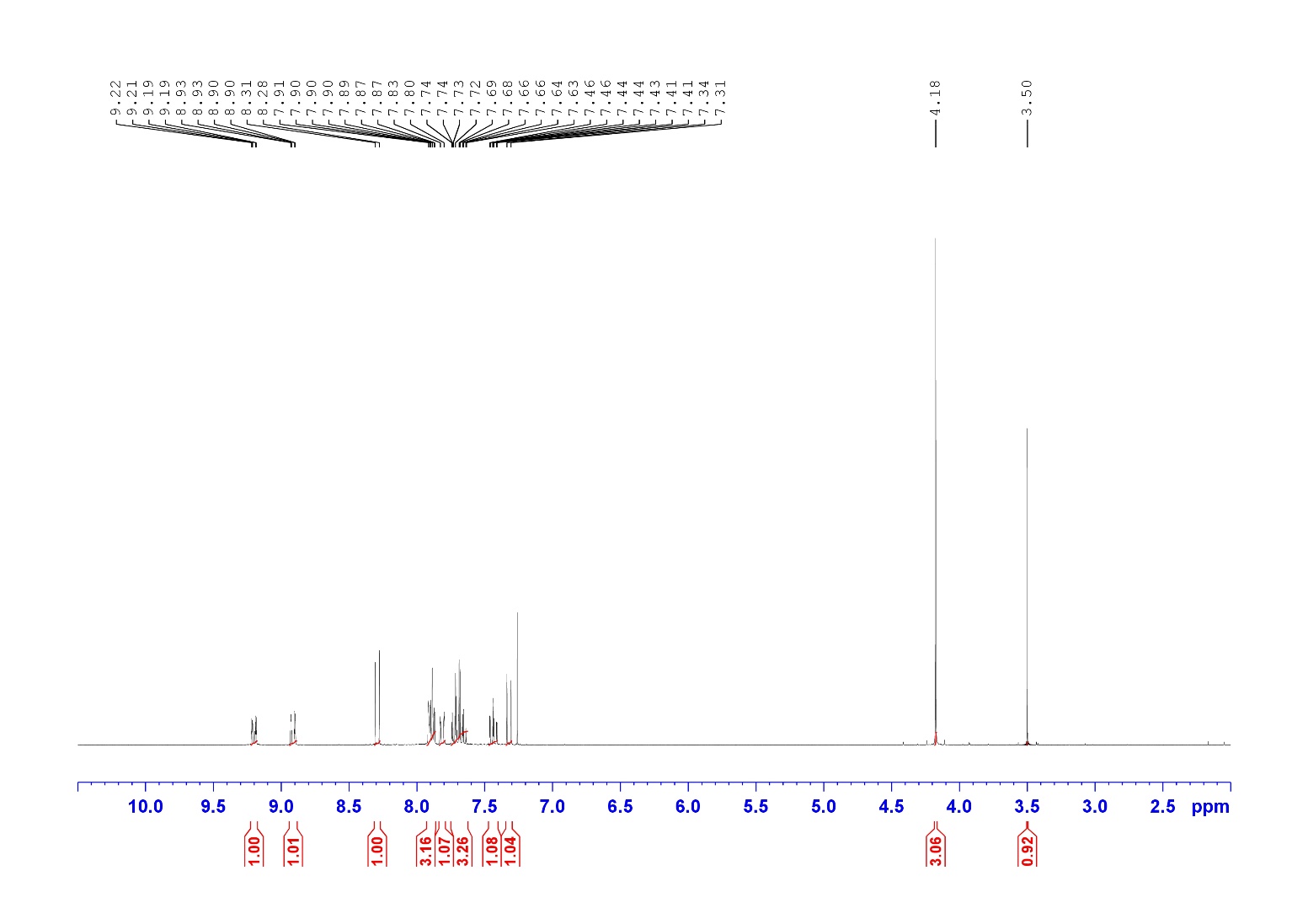

**Compound TMS-8**, ^1^H NMR (CDCl_3_)


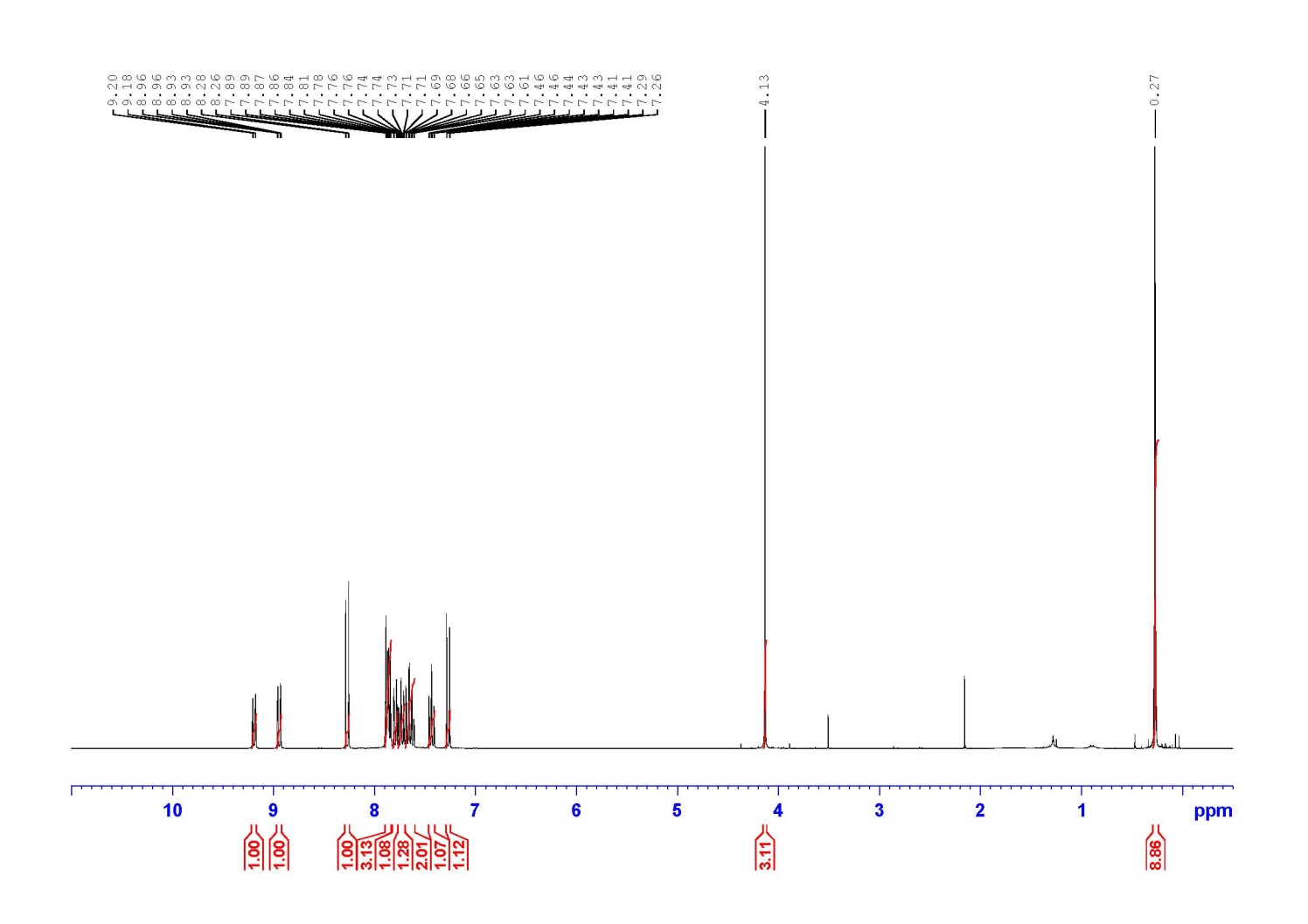

# ^13^C NMR (CDCl_3_)


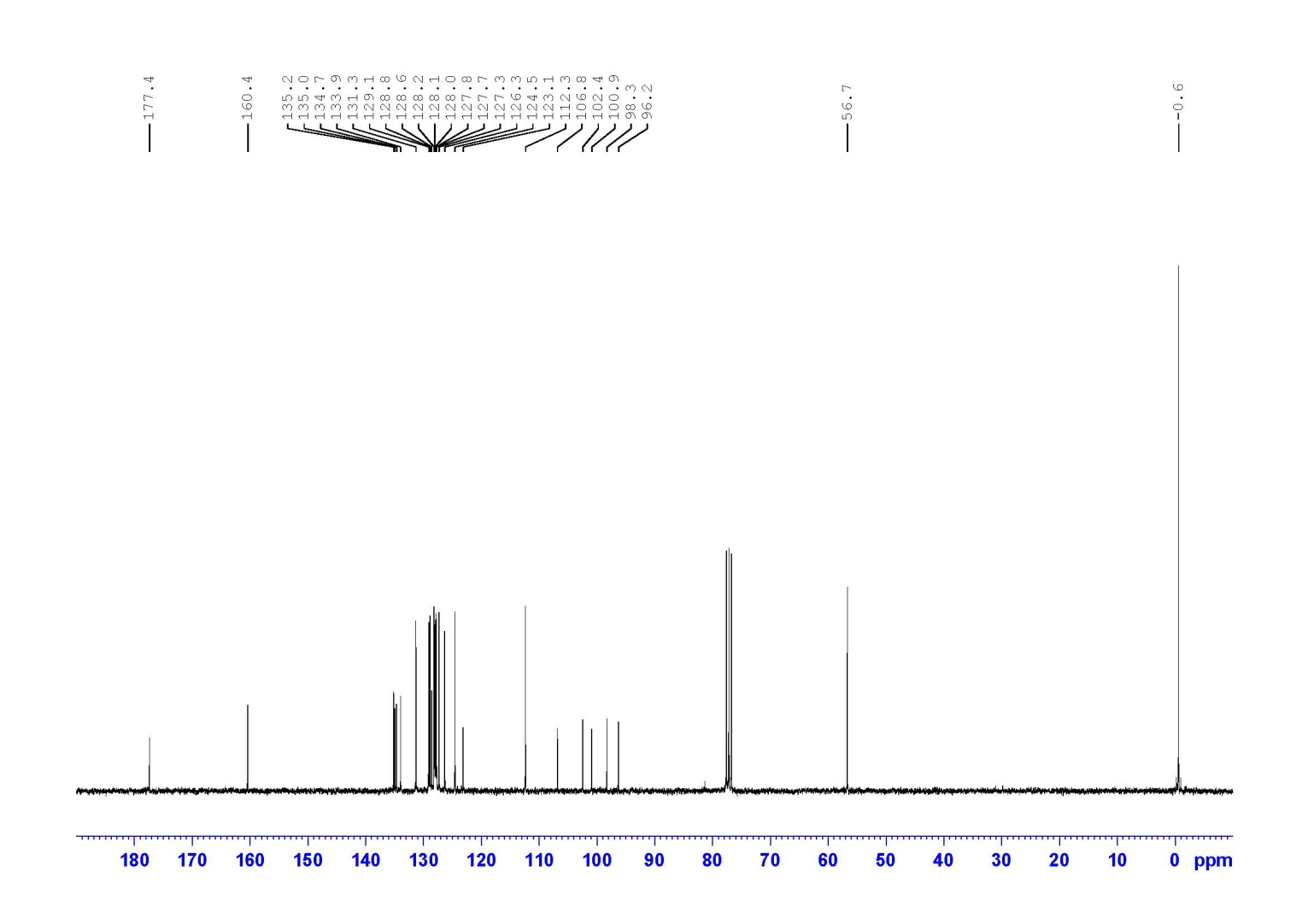


# Compound 9, ^1^H NMR (CDCl_3_)


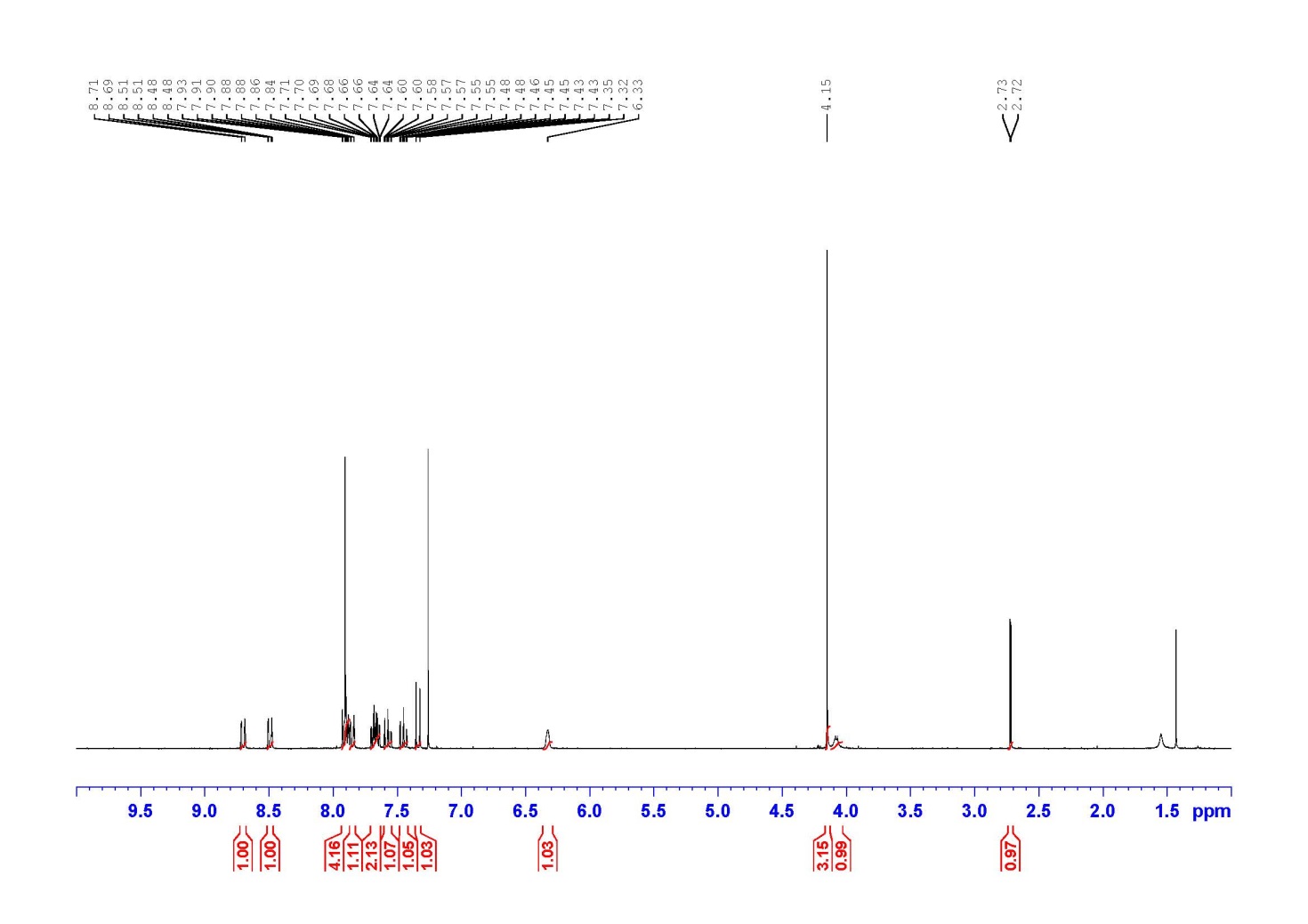


H_2_O

residual

cyclohexane

# ^13^C NMR (CDCl_3_)


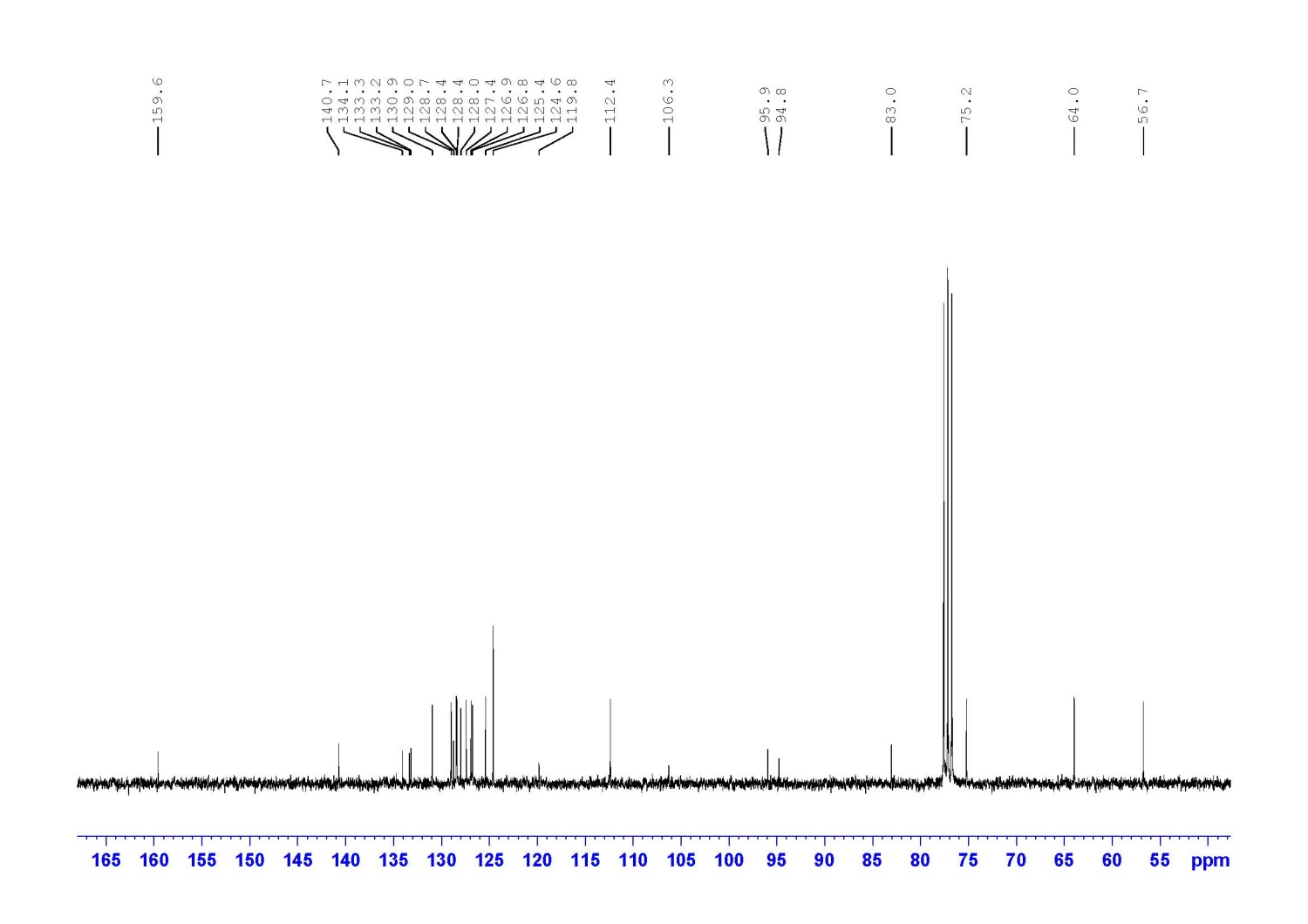


# Compound *rac*-10a, ^1^H NMR (CDCl_3_)


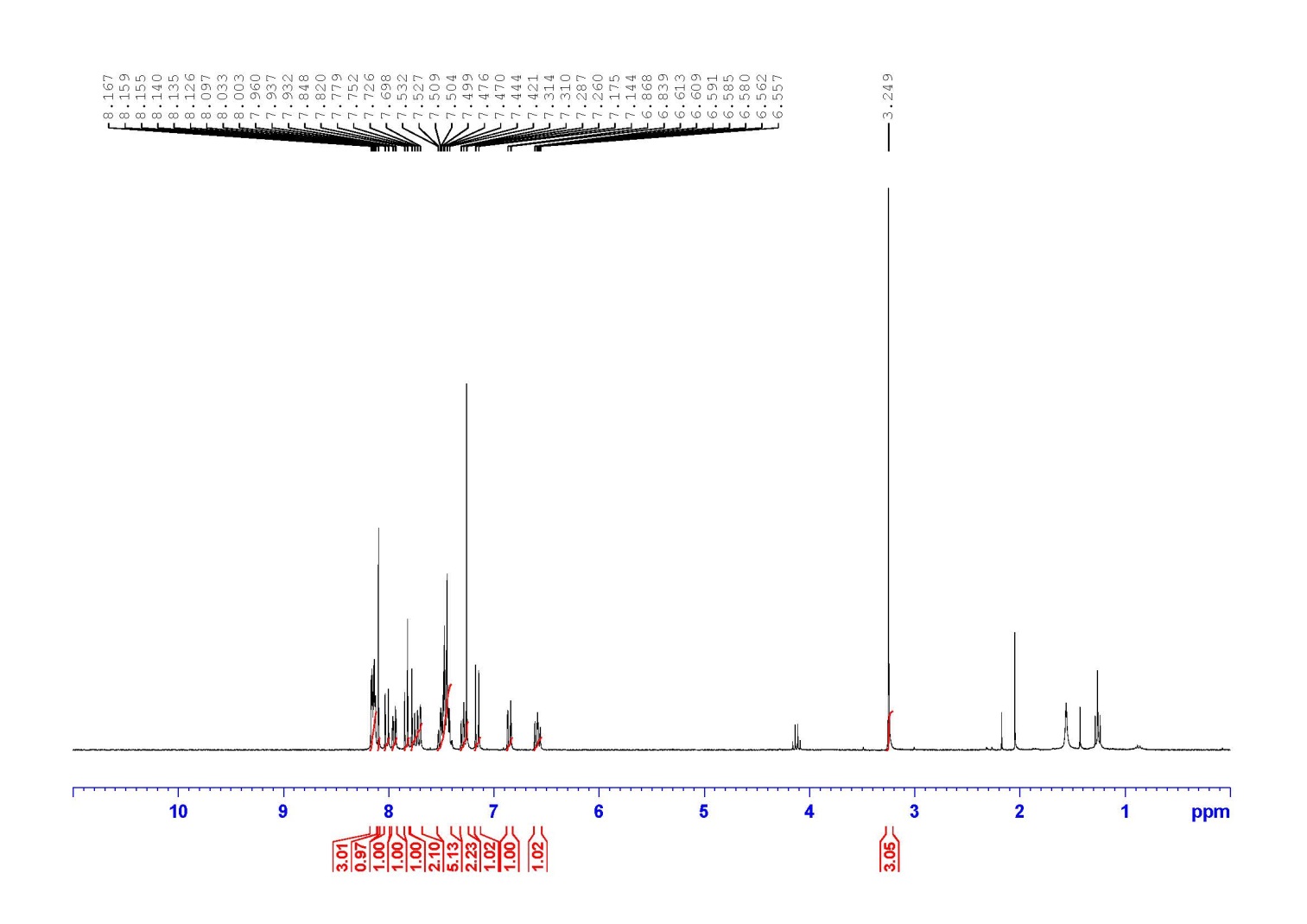

# ^13^C NMR (CDCl_3_)


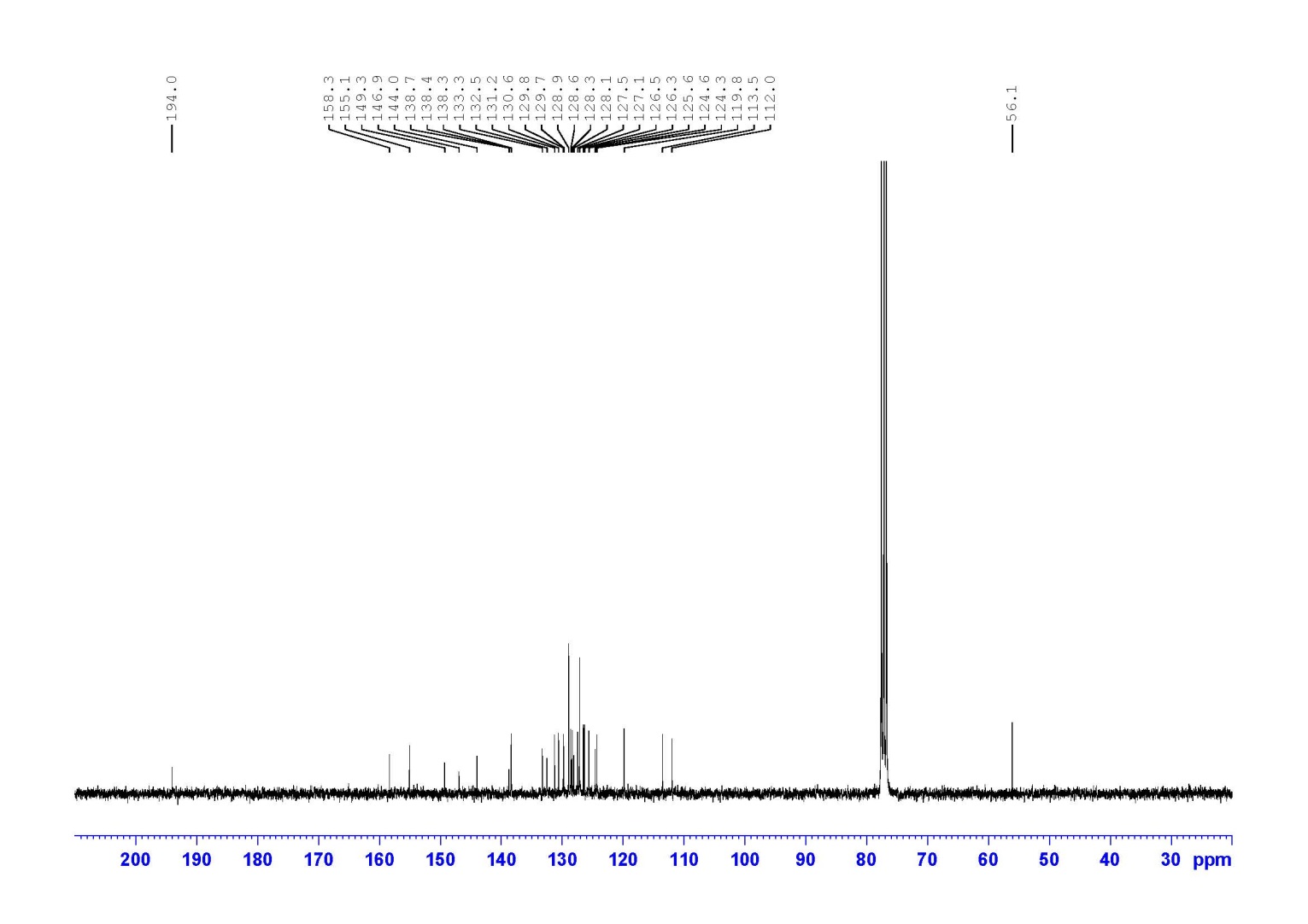


# Compound *rac*-10b, ^1^H NMR (CDCl_3_)


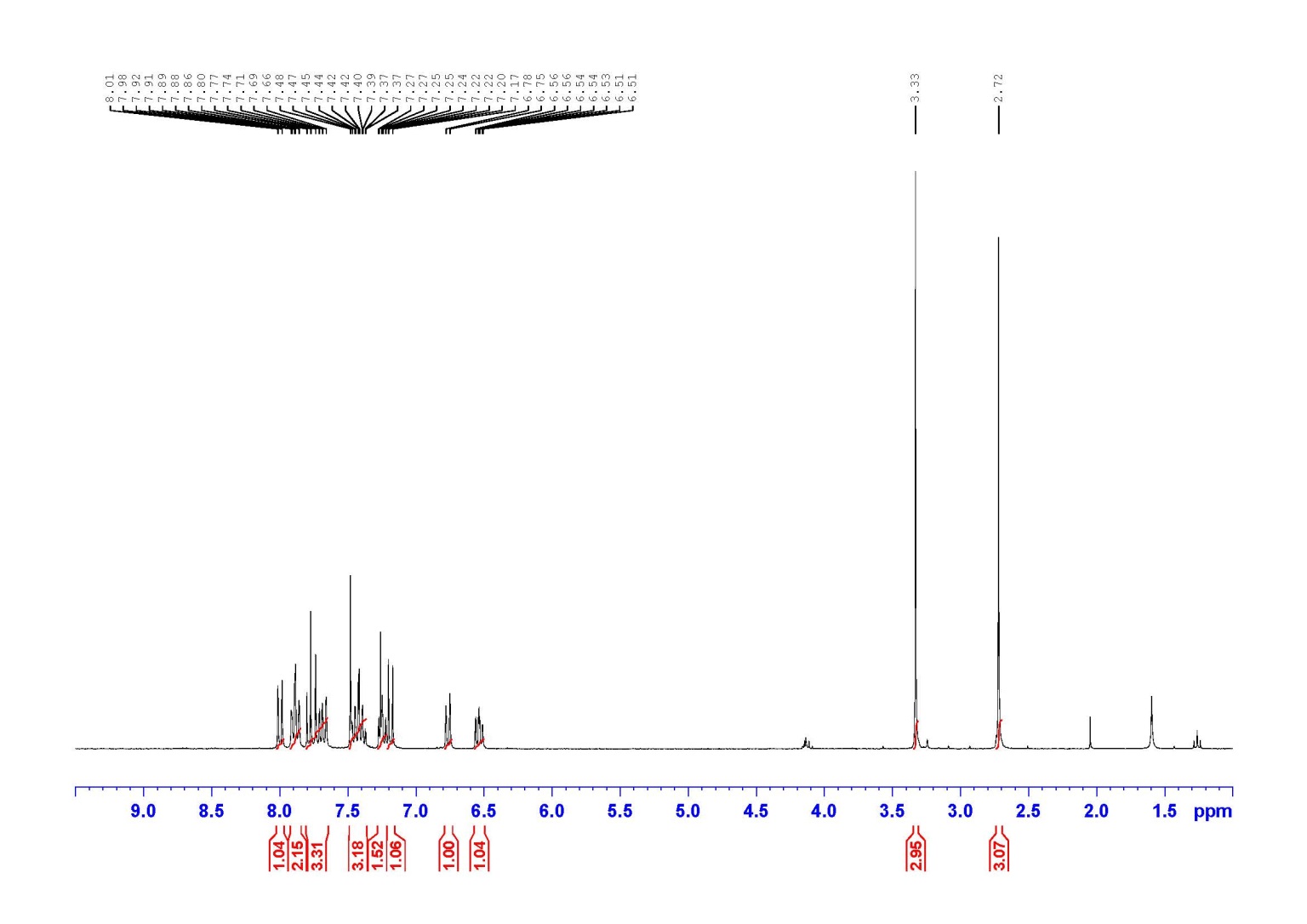

# ^13^C NMR (CDCl_3_)


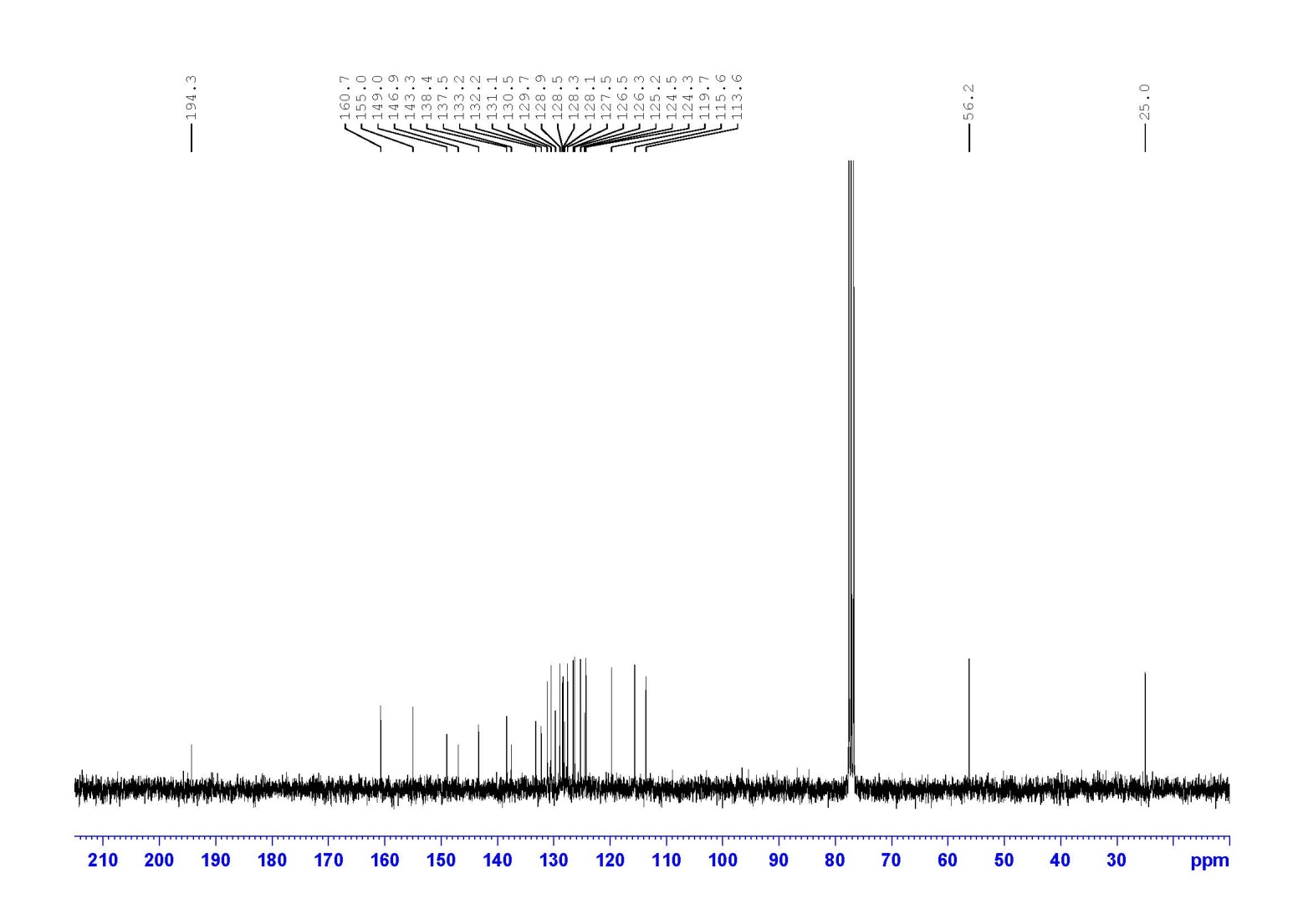


# Compound *rac*-10c, ^1^H NMR (CDCl_3_)


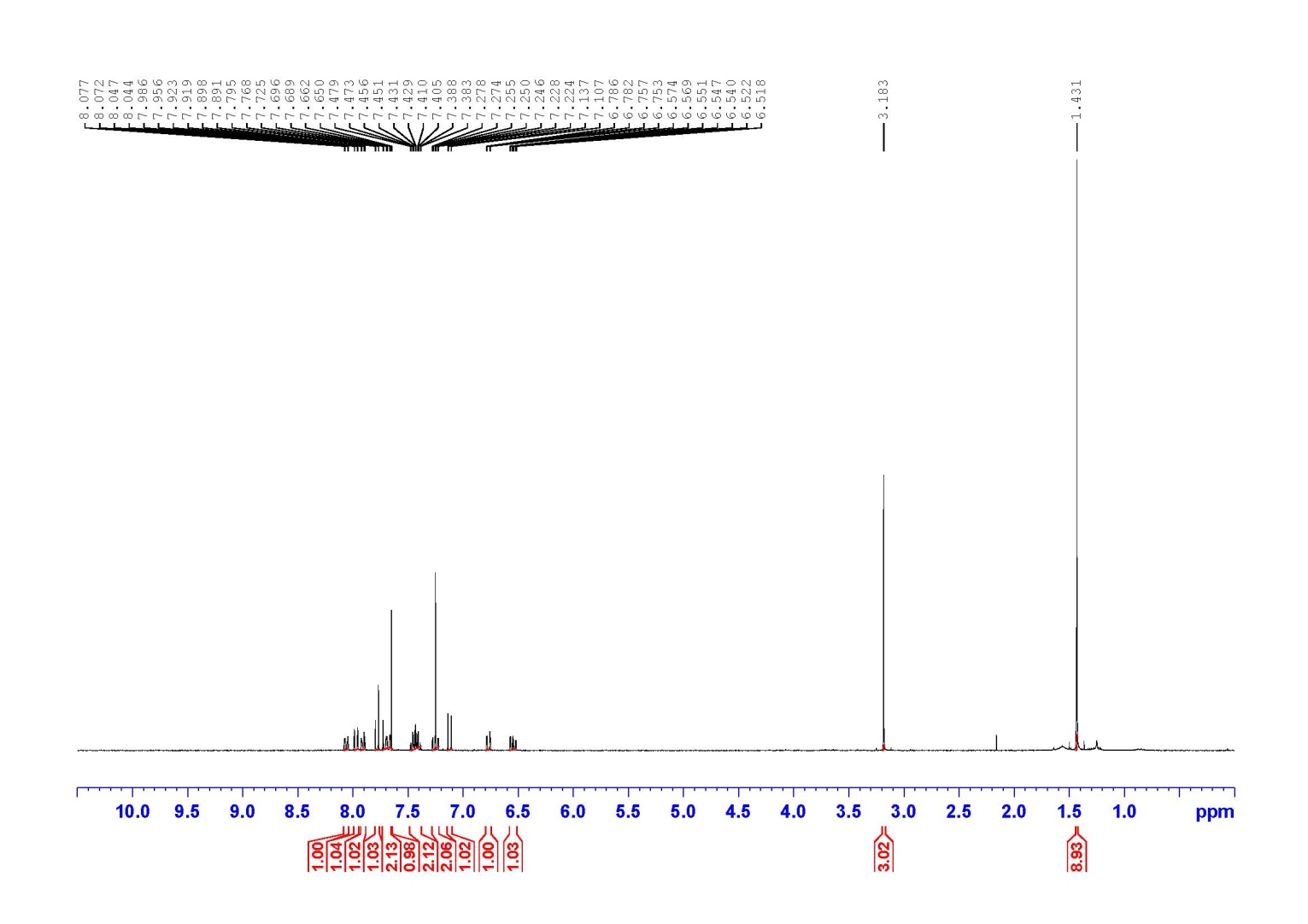

# ^13^C NMR (CDCl_3_)


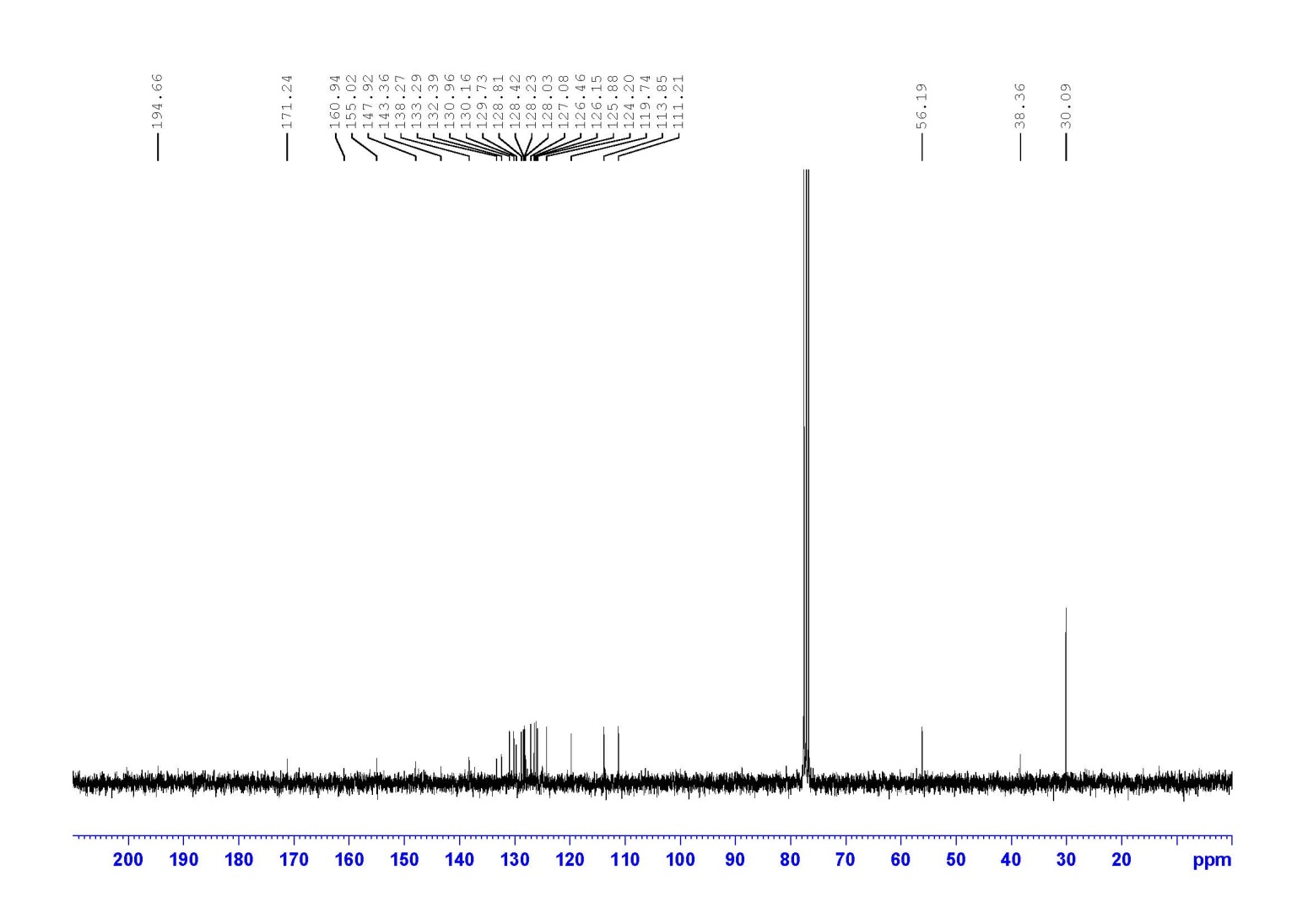


# Compound *rac*-10d, ^1^H NMR (CDCl_3_)


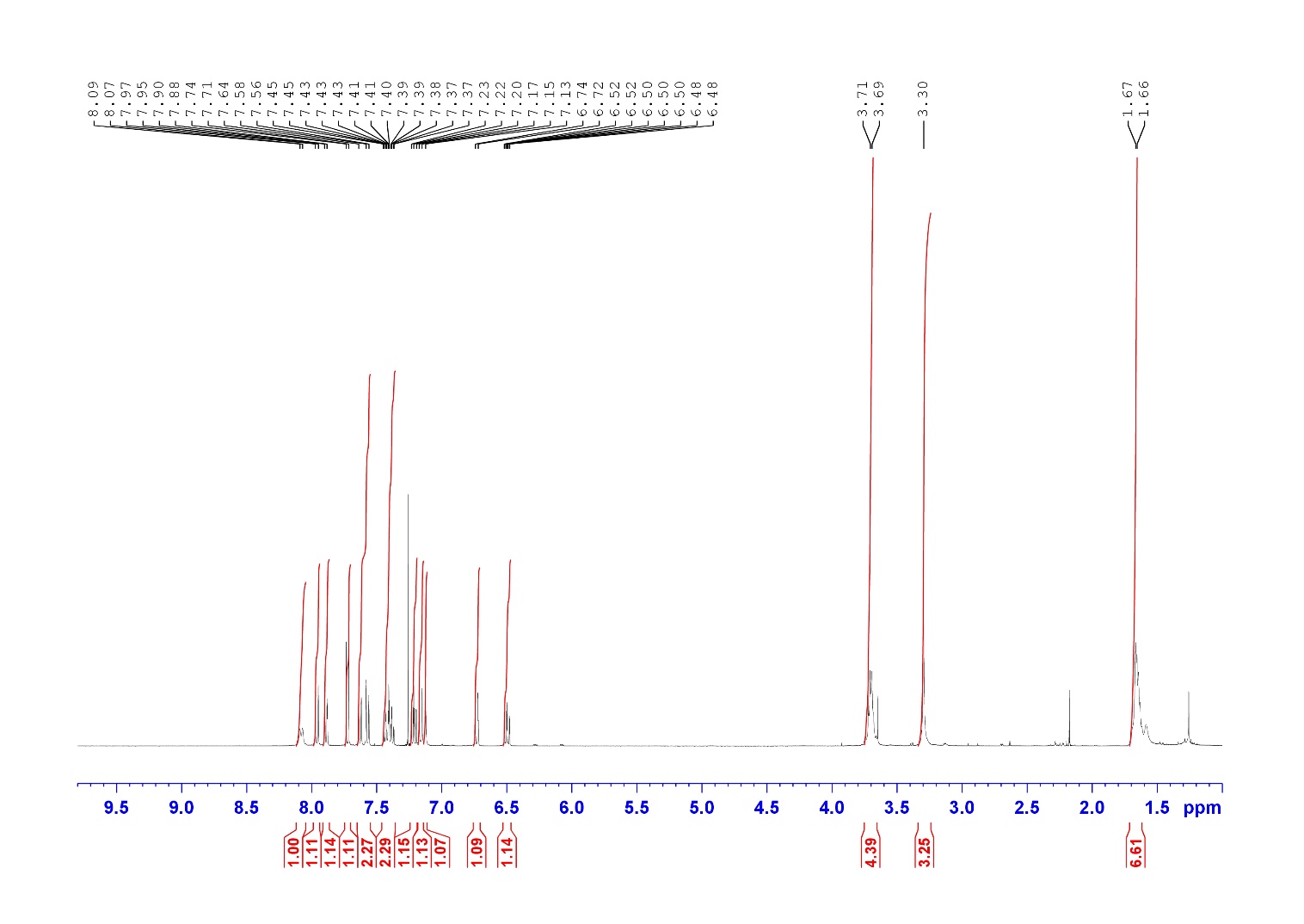

# ^13^C NMR (CDCl_3_)


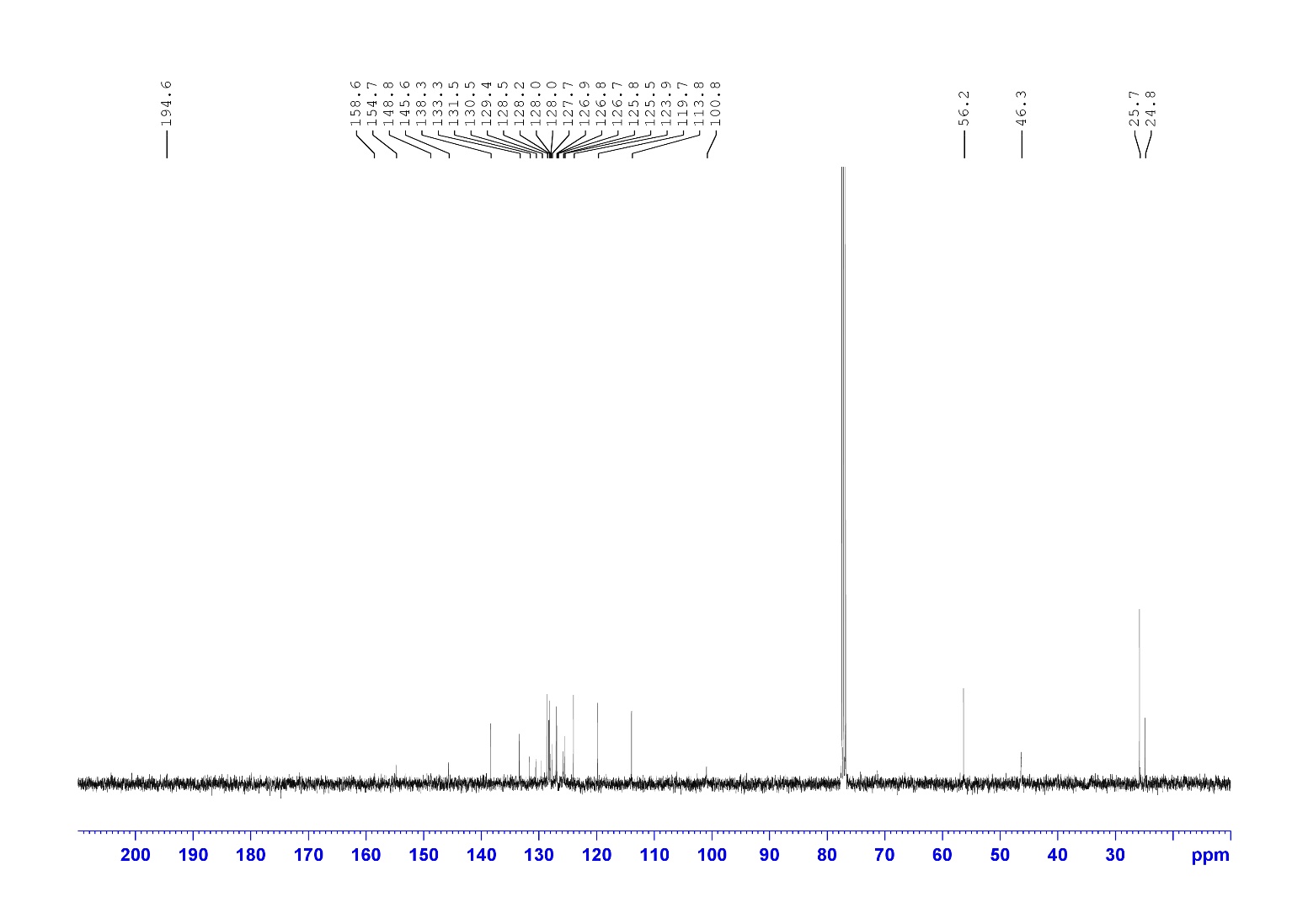


# Compound *rac*-10e, ^1^H NMR (CDCl_3_)


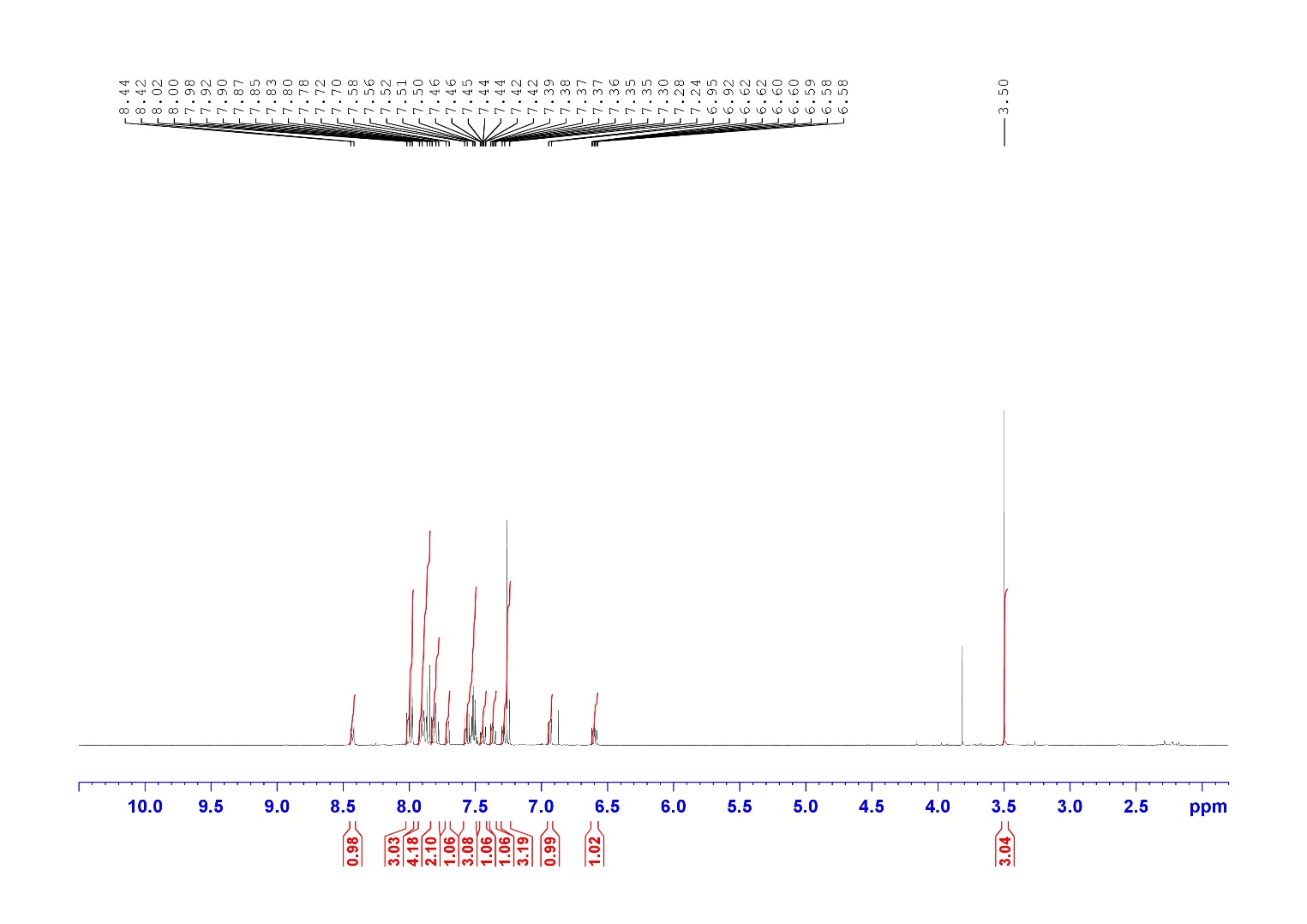

# ^13^C NMR (CDCl_3_)


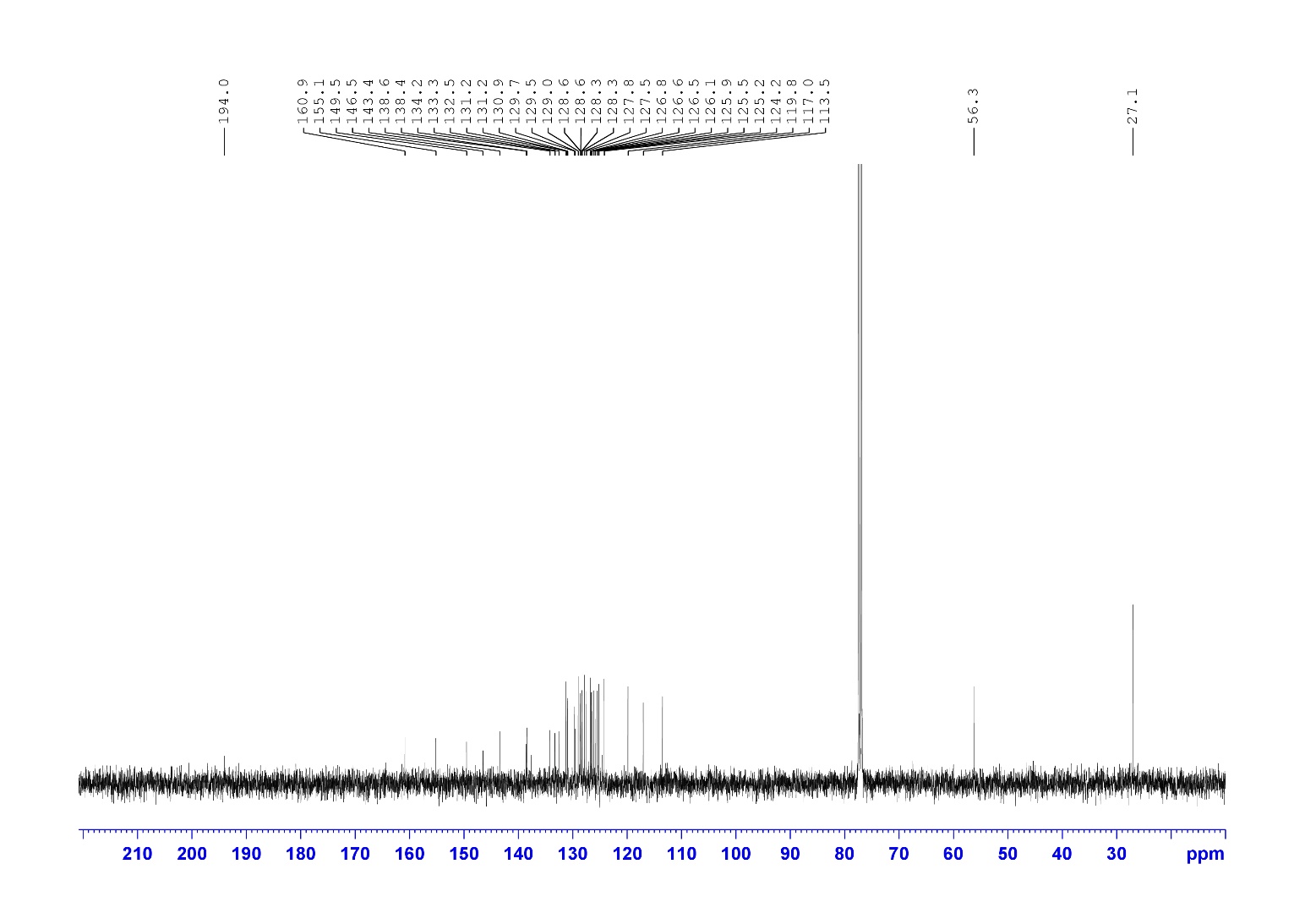


residual

cyclohexane
